# Methyltransferase complex subunit METTL3 maintains genome stability of erythroid cells via MTHFD1-mediated nucleotide biosynthesis

**DOI:** 10.1172/JCI196578

**Published:** 2026-03-10

**Authors:** Linlin Zhang, Huizhi Zhao, Shihui Wang, Xueting Wu, Donghao Liu, Hengchao Zhang, Qianqian Yang, Ying Cheng, Xiuyun Wu, Jiangwei Zhao, Shijie Zhang, Huan Zhang, Haojian Zhang, Qiaozhen Kang, Lixiang Chen, Xiuli An, Xiaoli Qu

**Affiliations:** 1 School of Life Sciences, Zhengzhou University, Zhengzhou, China.; 2 Department of Hematology, Institute of Hematology, Henan Provincial People’s Hospital, People’s Hospital of Zhengzhou University, Zhengzhou, China.; 3 The State Key Laboratory Breeding Base of Basic Science of Stomatology and Key Laboratory of Oral Biomedicine Ministry of Education, School and Hospital of Stomatology, Wuhan University, Wuhan, China.; 4 Laboratory of Membrane Biology, New York Blood Center, New York, New York, USA.

**Keywords:** Cell biology, Hematology, Epigenetics, Genetic instability

## Abstract

N^6^-methyladenosine (m^6^A) is a prevalent modification of mammalian mRNA. Increasing evidence has documented diverse roles of m^6^A in normal cell physiology and diseases. However, its functional role in erythropoiesis remains poorly understood. In this study, we found that deletion of *Mettl3* using the *EpoR-Cre* mouse led to microcytic/hypochromic anemia due to defective erythropoiesis along with impaired hemoglobin biosynthesis. Mechanically, *Mettl3* deficiency disrupted nucleotide biosynthesis, which induced DNA damage, leading to apoptosis of colony-forming unit–erythroid cells and cell-cycle arrest of erythroblasts. Integrated m^6^A-seq and RNA-seq analysis along with biochemical studies identified Mthfd1, a key enzyme involved in nucleotide biosynthesis, as a Mettl3 direct target gene. Furthermore, deletion of *Mettl3* led to decreased expression of *Mthfd1*, accompanied by a shortage of nucleotides deoxythymidine monophosphate and inosine monophosphate, in erythroid cells. Additionally, inhibition of METTL3 in human erythroid cells led to similar phenotypic and molecular changes, indicating a conserved role of METTL3 in human and murine erythropoiesis. Our findings have identified an METTL3-m^6^A-*MTHFD1* axis that plays a critical role in erythropoiesis by maintaining genome stability of erythroid cells via regulation of nucleotide biosynthesis. These findings provide important insights into the regulatory mechanisms of erythropoiesis and may have implications for underlying the mechanisms of anemias.

## Abstract 2

An unrecognized role of METTL3 in erythropoiesis by safeguarding genomic stability via regulating nucleotide biosynthesis through METTL3-m^6^A-MTHFD1 axis.

## Graphical abstract

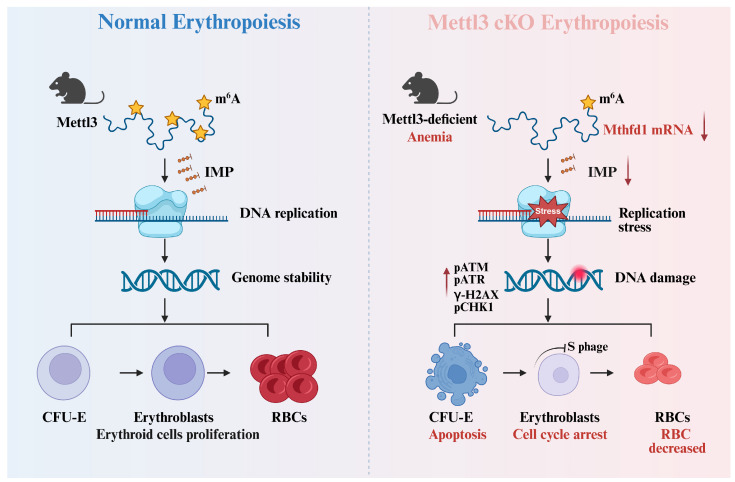

## Introduction

In adult humans, the body must produce, through a process termed erythropoiesis, approximately 200 billion new erythrocytes per day to maintain oxygen demand (1). During erythropoiesis, hematopoietic stem cells (HSCs) proliferate and differentiate progressively into burst-forming unit-erythroid (BFU-E) progenitors, colony-forming unit-erythroid (CFU-E) progenitors, proerythroblasts (ProEs), basophilic erythroblasts, polychromatic erythroblasts, and orthochromatic erythroblasts which extrude the nucleus to form reticulocytes (Retics). Retics mature into erythrocytes (2–5). The process of erythropoiesis is tightly regulated by multiple mechanisms. The well-established regulatory mechanisms include signaling transduction mediated by erythropoietin (EPO) and erythropoietin receptor (EPOR) (6), transcriptional regulation by master transcription factors GATA1 and KLF1 (7, 8), epigenetic regulation via DNMT-mediated DNA methylation (9, 10), TET2/TET3-mediated DNA demethylation (11–14), histone deacetylase–mediated histone modification (15–17). RNA modification is another form of epigenetic regulation (18, 19). However, in contrast to the extensive studies cited here, our knowledge of the role of RNA modification in erythropoiesis is very limited.

RNA modifications mainly include methylation, such as N^6^-methyladenosine (m^6^A), N^1^-methyladenosine, and 5-methylcytosine (20). m^6^A is the most abundant modification of mRNA. In the past few years, an increasingly important role of m^6^A in normal cell physiology and disease has been documented (19–21). m^6^A regulates transcriptional processing (22), nuclear export (23), stabilization (24), and translation of the mRNA (25). m^6^A is deposited onto mRNAs by a methyltransferase complex, composed of methyl transferase-like 3 (METTL3), methyltransferase-like 14, Wilms tumor–associated protein, and other accessory proteins (26). METTL3 is the only catalytic component of the complex (27). A previous study showed that whole-body Mettl3 knockout mice exhibited early embryonic lethality (21). In the context of hematopoiesis, it has been reported that Mettl3 is essential for hematopoietic cell development and function (28–31). A previous study showed that m^6^A regulates erythroid gene expression in erythroid cells and that knocking down *METTL3* in human CD34^+^ cells significantly decreased erythroid cell production (32). However, the role of Mettl3 in erythropoiesis in vivo and its underlying mechanisms have yet to be defined.

In the present study, we deleted *Mettl3* in erythroid cells, using the *EpoR-Cre* mouse we recently generated (33). The conditional *Mettl3*-knockout mice exhibited microcytic hypochromic anemia due to defective erythropoiesis along with impaired hemoglobin (HGB) biosynthesis. Mechanistically, *Mettl3* deficiency led to apoptosis of CFU-E cells and cell-cycle arrest of erythroblasts. At the molecular level, we found that Mthdf1, a key enzyme involved in nucleotide biosynthesis (34), was a direct target of Mettl3 and that deletion of *Mettl3* led to decreased expression of Mthfd1. Consequently, the decreased expression of Mthfd1 resulted in defective DNA synthesis, as demonstrated by decreased levels of deoxythymidine monophosphate (dTMP) and inosine monophosphate (IMP) in erythroid cells, inducing DNA damage that triggered apoptosis in CFU-E cells and cell-cycle arrest in ProEs. Furthermore, inhibition of METTL3 in human erythroid cells led to similar phenotypic and molecular changes as observed in mice. Together, our findings have uncovered an unrecognized role of METTL3 in erythropoiesis by safeguarding genomic stability via regulating nucleotide biosynthesis through a METTL3/m^6^A/*MTHFD1* axis.

## Results

### Deletion of Mettl3 using EpoR-tdTomato-Cre led to microcytic hypochromic anemia in mice.

**Figure 1:**
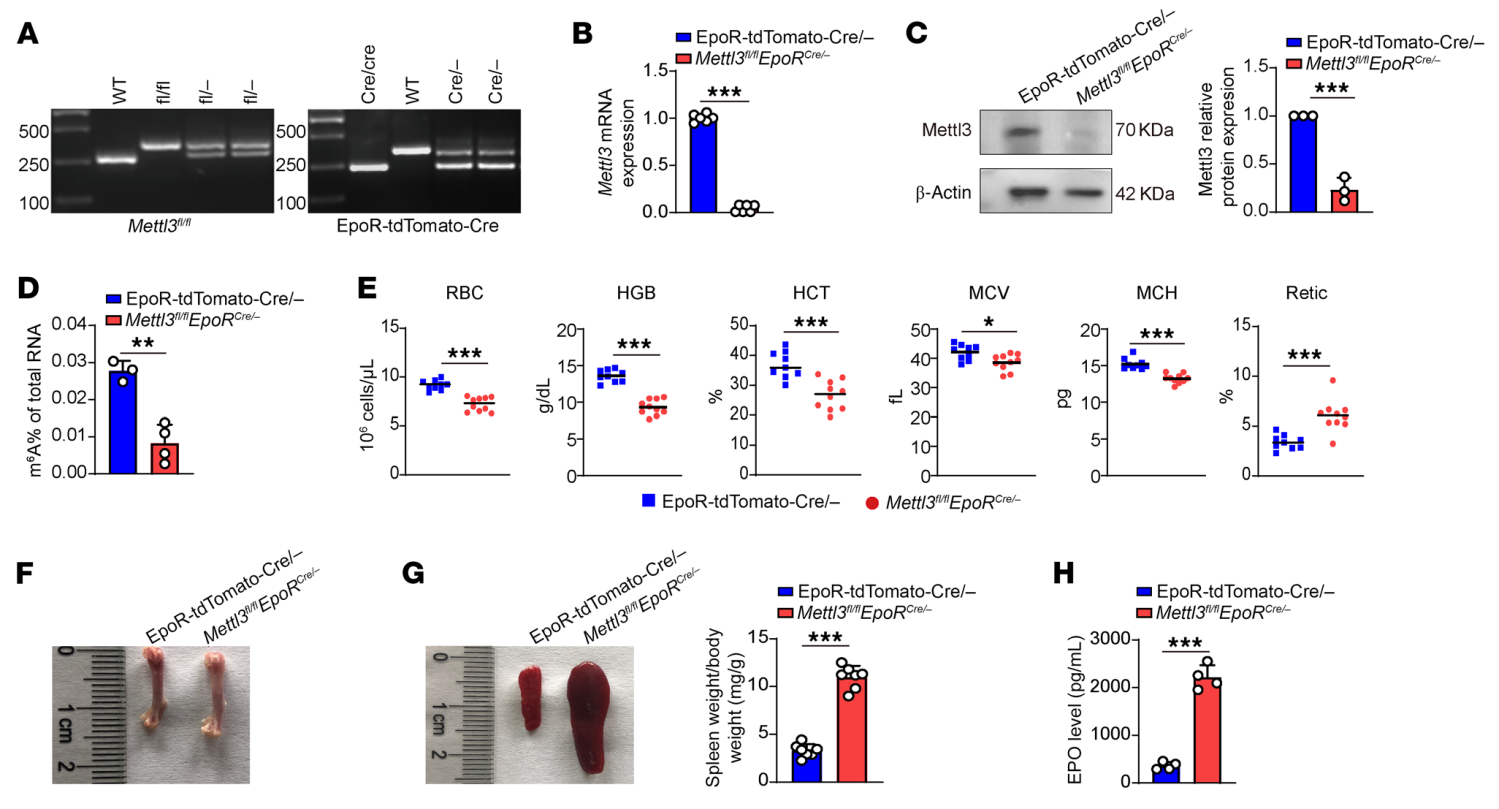
*Mettl3* deletion using *EpoR-tdTomato-Cre* led to anemia in mice. (**A**) PCR genotyping analysis of *EpoR-tdTomato-Cre* and *Mettl3*-floxed mice. (**B**) *Mettl3* mRNA expression level measured by qRT-PCR and normalized to β-actin in erythroid cells (*n* = 6/group). (**C**) Representative Western blot and quantitative analysis of Mettl3 knockout efficiency in mice erythroid cells (*n* = 3/group). (**D**) Quantification of m^6^A in total RNA of erythroid cells via colorimetric assay (*n* = 3–4/group). (**E**) Complete blood cell count analysis of peripheral blood (*n* = 9–10/group). (**F**) Representative images of dissected femurs. (**G**) Representative images of spleens and quantification of splenomegaly by spleen-to-body weight ratio (*n* = 7/group). (**H**) Mice serum EPO levels as measured by ELISA (*n* = 4/group). Data are presented as the mean ± SD. Comparisons between 2 groups were performed using an unpaired 2-tailed Student’s *t* test. *P < 0.05, **P < 0.01, ***P < 0.001. MCH, mean cellular hemoglobin; MCV, mean corpuscular volume.

A previous study showed that knockdown of *METTL3* in CD34^+^ cells impaired human erythropoiesis in vitro (32). To investigate the functional role of Mettl3 during erythropoiesis in vivo, we crossed *Mettl3^fl/fl^* mice (35) with *EpoR-tdTomato-Cre* mice, which efficiently recombine in erythroid cells and erythroblastic island (EBI) macrophages (33). The genotype was assessed by PCR (Figure 1A). As shown in Supplemental Figure 1, A–C, embryos of the *Mettl3^fl/fl^ EpoR^Cre/cre^* were smaller and appeared paler at the E14.5–E16.5 stages, yet their numbers remained consistent with Mendelian ratios (Supplemental Figure 1D; supplemental material available online with this article; https://doi.org/10.1172/JCI196578DS1). However, no individuals survived by 3 weeks after birth, suggesting perinatal lethality.

To investigate the cause of lethality in the *Mettl3^fl/fl^ EpoR^Cre/cre^* mice, we analyzed E16.5 fetal liver cells and observed a significant reduction in the total cellularity along with exhaustion of long-term HSCs and a severe decrease in erythroid cells (Supplemental Figure 1, E–G). For subsequent studies, *Mettl3^fl/fl^ EpoR^Cre/–^* mice were used. As expected, *Mettl3* was completely deleted in erythroid cells, as shown by the absence of its mRNA (Figure 1B) and protein (Figure 1C). Consequently, the global m^6^A level was significantly decreased in the enriched erythroid cells from the *Mettl3^fl/fl^ EpoR^Cre/–^* mice (Figure 1D). Analyses of peripheral blood revealed microcytic hypochromic anemia of the *Mettl3^fl/fl^ EpoR^Cre/–^* mice, as demonstrated by decreases in RBC counts, HGB, hematocrit (HCT), mean corpuscular volume, and mean cellular hemoglobin, along with increase in Retics (Figure 1E). Consistent with the decreased mean corpuscular volume, peripheral blood smear also revealed decreased size of mature erythrocytes of *Mettl3^fl/fl^ EpoR^Cre/–^* mice compared with control mice (Supplemental Figure 2A). Because microcytic hypochromic anemia is associated with impaired HGB synthesis (36), we examined the expression of genes involved in HGB biosynthesis and found that *Alas2* and *Fech*, the key genes involved in heme biosynthesis, as well as α- and β-globin genes were significantly decreased in *Mettl3*-deficient erythroblasts compared with control erythroblasts (Supplemental Figure 2, B–G).

To determine if Mettl3 directly regulates HGB synthesis via m^6^A, we performed methylated RNA IP quantitative PCR (MeRIP-qPCR) using erythroblasts. Supplemental Figure 2, H–K, shows that high levels of m^6^A were detected on *Alas2*, *Fech*, *Hba*, and *Hbb* in control cells. Notably, Mettl3 deletion led to a reduction of m^6^A levels on *Fech*, *Hba*, and *Hbb*, but not *Alas2*. Moreover, the femur of *Mettl3^fl/fl^ EpoR^Cre/–^* mice was markedly paler (Figure 1F) and the spleen size of *Mettl3^fl/fl^ EpoR^Cre/–^* mice was significantly bigger (Figure 1G). Consistent with splenomegaly, *Mettl3*-deficient mice exhibited significantly increased blood EPO levels (Figure 1H) and an expansion of erythroblasts in the spleen (Supplemental Figure 3) compared with control mice. These findings demonstrate that deletion of Mettl3 using *EpoR-tdTomato-Cre* in mice resulted in compensatory microcytic hypochromic anemia.

### Deletion of Mettl3 in erythroid cells resulted in impaired erythropoiesis.

**Figure 2:**
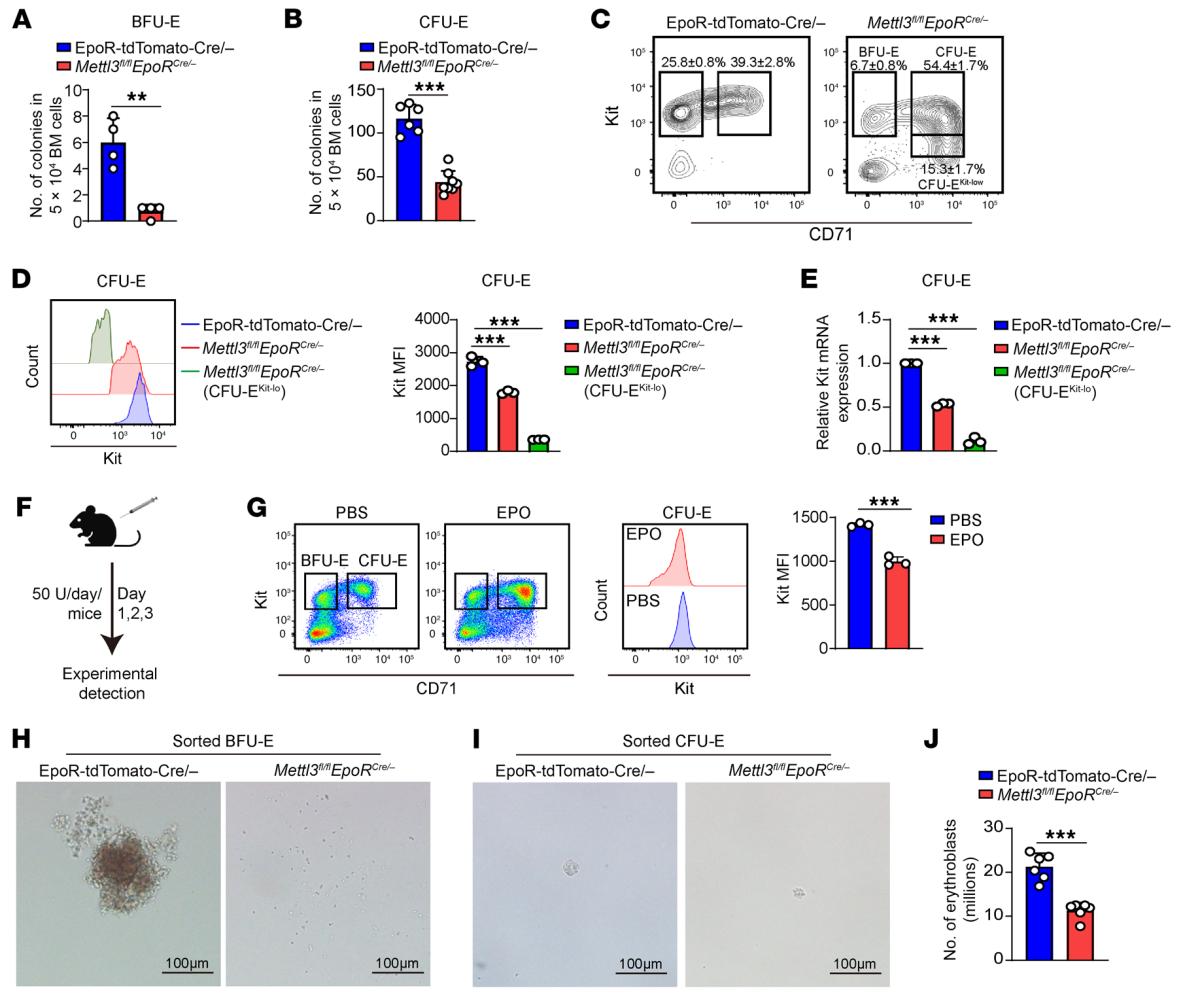
*Mettl3* deficiency in erythroid cells impaired mouse BM erythropoiesis. (**A**) Quantification of BFU-E colonies in 5 × 10^4^ BM cells (*n* = 4/group). (**B**) Quantification of CFU-E colonies in 5 × 10^4^ BM cells (*n* = 6–8/group). (**C**) Flow cytometric analysis of erythroid progenitors. Contour plots display gated 7AAD^–^Lin^–^CD16/32^–^CD41^–^CD34^–^Sca1^–^ cells, with BFU-E (Kit^+^CD71^–^) and CFU-E (Kit^+^CD71^+^) populations delineated. CFU-E^Kit-lo^ indicate the CFU-E subpopulation with reduced Kit expression in *Mettl3*-deficient mice. (**D**) Flow cytometric analysis and quantification of Kit expression on CFU-E cells (*n* = 3/group). (**E**) Quantification of *Kit* mRNA expression in control and *Mettl3*-deficient CFU-E cells by qRT-PCR (*n* = 3/group). (**F**) Schematic of the EPO administration in mice. (**G**) Analysis of Kit expression in CFU-E cells by flow cytometry at 3 days after EPO injection (*n* = 3/group). (**H**) Representative images of sorted BFU-E colonies from mice BM. Scale bar: 100 μm. (**I**) Representative images of sorted CFU-E colonies from mice BM. Scale bar: 100 μm. (**J**) Quantification of mice BM erythroblasts numbers (*n* = 6/group). Data are presented as the mean ± SD. Comparisons between 2 groups were performed using an unpaired 2-tailed Student’s *t* test. A 2-way ANOVA with Tukey’s post hoc test was used to calculate statistical significance among multiple groups. **P < 0.01, ***P < 0.001.

To investigate the cellular mechanisms for the anemia in *Mettl3^fl/fl^ EpoR^Cre/–^* mice, we first assessed erythroid progenitors in the BM by erythroid colony-forming assay using total BM cells and found that both BFU-E (Figure 2A) and CFU-E (Figure 2B) colonies were significantly decreased in the BM cells of the *Mettl3^fl/fl^ EpoR^Cre/–^* mice compared with control mice. We further analyzed erythroid progenitors by flow cytometry in which BFU-E and CFU-E cells were defined as Kit^+^CD71^–^ and Kit^+^CD71^+^, respectively (33). Notably, the expression levels of Kit on *Mettl3*-deficient CFU-E cells were significantly decreased, with a subset of CFU-E cells exhibiting drastic reduction. Based on the expression levels of Kit, the *Mettl3*-deficient CFU-E cells could be divided into 2 populations, which we referred to as CFU-E and CFU-E^Kit-lo^ cells, respectively (Figure 2C and Supplemental Figure 4). Quantitative analysis of MFI revealed that *Mettl3* deletion reduced the Kit expression by approximately 34% on CFU-E and approximately 89% on CFU-E^Kit-lo^ populations (Figure 2D). In parallel with decreased surface expression, the mRNA levels of *Kit* were also significantly decreased (Figure 2E).

Given that EPO has been reported to suppress Kit expression in erythroid cell lines (37) and that EPO levels were increased in *Mettl3*-deficient mice, we examined the effect of EPO on Kit expression on erythroid progenitors in vivo. We found that EPO injection led to an approximately 29% reduction of Kit expression on CFU-E cells (Figure 2, F and G), indicating the decreased expression of Kit on *Mettl3*-deficient CFU-E cells could be due to increased EPO levels; the reason for the approximately 89% decrease on CFU-E^Kit-lo^ cells remains unclear.

We further sorted BFU-E and CFU-E cells and examined their colony-forming abilities and found that the BFU-E colonies and CFU-E colonies formed by the sorted *Mettl3*-deficient BFU-E cells and CFU- E cells were much smaller than the corresponding control BFU-E and CFU-E cells (Figure 2, H and I). Furthermore, the sorted CFU-E^Kit-lo^ cells could not form colonies. We stained erythroblasts using Ter119, CD44, and forward scatter as parameters (3) and found that erythroblasts were significantly decreased in the BM of the *Mettl3^fl/fl^ EpoR^Cre/–^* mice (Figure 2J and Supplemental Figure 5). These findings demonstrate that deletion of *Mettl3* using the *EpoR-tdTomato-Cre* significantly impaired erythropoiesis.

We recently showed that in addition to erythroid cells, EpoR is also expressed in EBI macrophages (33). To examine whether deletion of *Mettl3* in EBI macrophages contributes to the impaired erythropoiesis of the *Mettl3^fl/fl^ EpoR^Cre/–^* mice, we selectively deleted *Mettl3* in macrophages by crossing *CD169-tdTomato-Cre* mice we recently generated (data not shown) with the *Mettl3^fl/fl^* mice to generate *Mettl3^fl/fl^ CD169^Cre/–^* mice (Supplemental Figure 6A). As shown in Supplemental Figure 6B, *Mettl3* was deleted in the sorted F4/80^+^CD169-tdTomato EBI macrophages. Notably, *Mettl3^fl/fl^ CD169^Cre/–^* mice did not exhibit significant changes in RBCs, HGB, HCT, mean cellular hemoglobin, and Retics (Supplemental Figure 6C). Furthermore, no changes in the percentages of EBI macrophages (Supplemental Figure 6D) and erythroid cells (Supplemental Figure 6E) were detected in the BM of *Mettl3^fl/fl^ CD169^Cre/–^* mice compared with control mice. These findings indicate that deletion of *Mettl3* in macrophages did not affect EBI macrophages and did not contribute to the anemia and impaired erythropoiesis of the *Mettl3^fl/fl^ EpoR^Cre/–^* mice.

In addition to erythroid cells and EBI macrophages, we also reported that EpoR is expressed in HSCs (33). We therefore examined whether *EpoR^Cre/–^* could delete *Mettl3* in HSCs and found that the mRNA levels of *Mettl3* in lineage^–^Sca-1^+^Kit^+^ cells from the *Mettl3^fl/fl^ EpoR^Cre/–^* mice were decreased by only about 50% (Supplemental Figure 7A), indicating *EpoR^Cre/–^* could not efficiently delete *Mettl3* in HSCs. Consequently, no significant changes in hematopoietic stem and progenitor cells (HSPCs) were detected between the *Mettl3^fl/fl^ EpoR^Cre/–^* and control mice (Supplemental Figure 7, B and C). Taken together, the anemia of the *Mettl3^fl/fl^ EpoR^Cre/–^* mice was mainly attributed to the deletion of *Mettl3* in erythroid cells.

### Mettl3 deletion led to apoptosis of CFU-E cells and cell-cycle arrest of erythroblasts, due to defective DNA synthesis.

**Figure 3:**
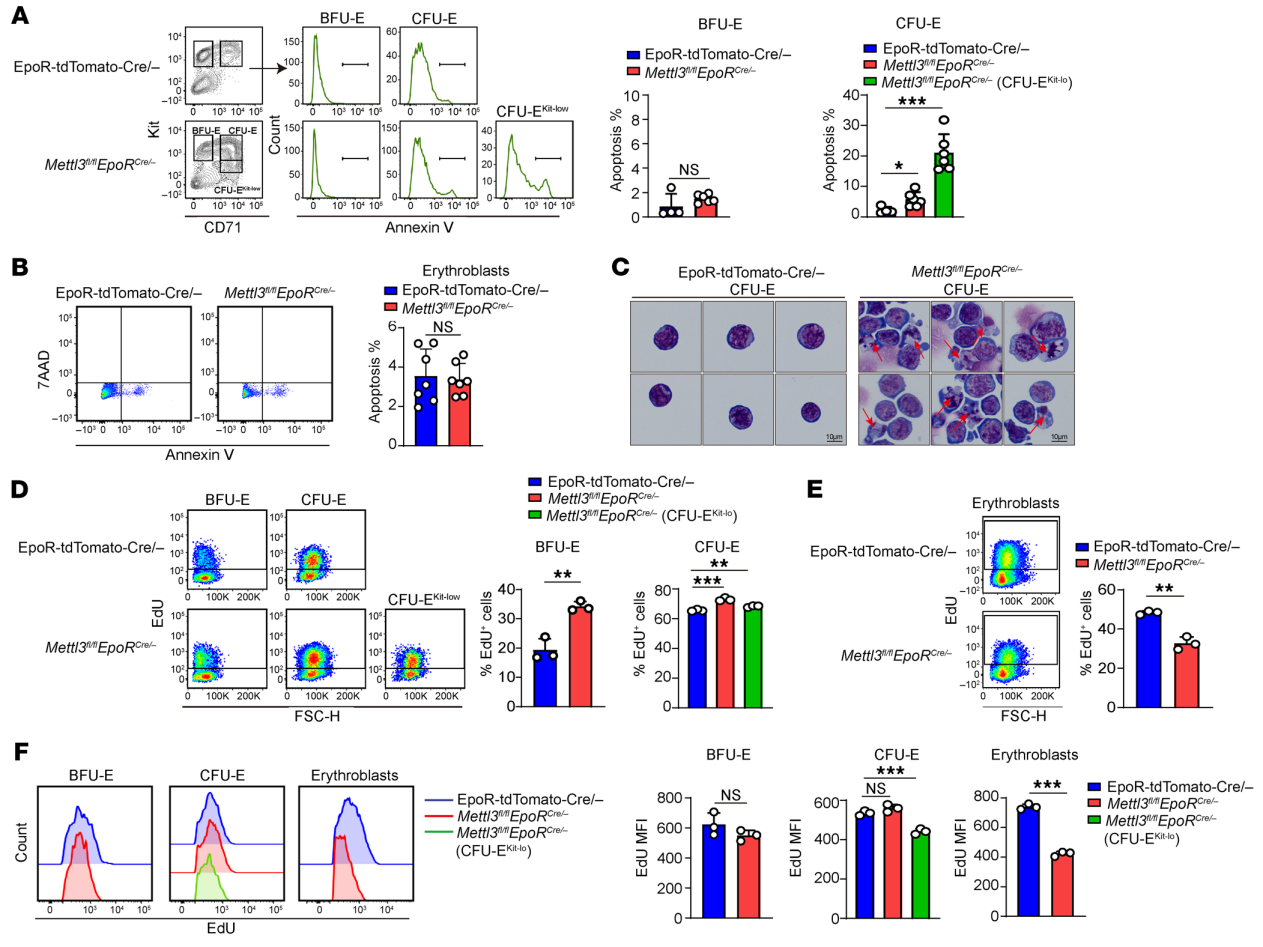
*Mettl3* deletion induced apoptosis of CFU-E and cell-cycle arrest of erythroblasts via impaired DNA synthesis. (**A**) Flow cytometric analysis of apoptosis in BFU-E and CFU-E erythroid progenitors using annexin V and 7AAD staining (*n* = 4–6/group). (**B**) Quantification of apoptotic BM erythroblasts by flow cytometry (*n* = 7/group). (**C**) Representative cytospin images of sorted CFU-E cells. Red arrows indicate apoptotic CFU-E cells. Scale bar: 10 μm. (**D**) Flow cytometric analysis of EdU^+^ cells in BFU-E and CFU-E cells (*n* = 3/group). (**E**) Flow cytometric analysis of EdU^+^ cells in erythroblasts (*n* = 3/group). (**F**) Quantification of S-phase MFI of BFU-E cells, CFU-E cells, and erythroblasts (*n* = 3/group). Data are presented as the mean ± SD. Comparisons between 2 groups were performed using an unpaired 2-tailed Student’s - test. A 2-way ANOVA with Tukey’s post hoc test was used to calculate statistical significance among multiple groups. *P < 0.05, **P < 0.01, ***P < 0.001. FSC-H, forward scatter height.

The decreases in erythroid progenitors or erythroblasts can be attributed to increases in apoptosis or/and decreases in cell proliferation. We first assessed apoptosis using annexin V and 7AAD staining. Figure 3A shows that although no significant difference was noted between control and *Mettl3*-deficient BFU-E cells, *Mettl3*-deficient CFU-E cells, particularly the CFU-E^kit-lo^ cells, exhibited an increased percentage of apoptotic cells. No differences in apoptosis were noted between control and *Mettl3*-deficient erythroblasts (Figure 3B). Consistent with flow cytometry analyses, cytospin analyses of the sorted total *Mettl3*-deficient CFU-E cells exhibited changes that resembled characteristic morphological changes of apoptosis, such as membrane blebbing and nuclear fragments (Figure 3C). In contrast, *Mettl3*-deficient BFU-E cells (Supplemental Figure 8A) and erythroblasts (Supplemental Figure 8B) appeared morphologically normal. These findings indicate that deficiency of *Mettl3* led to selective apoptosis of CFU-E cells.

We next assessed cell proliferation by in vivo 5-ethynyl-2′-deoxyuridine (EdU) incorporation assay (38). We found that while *Mettl3*-deficient BFU-E and CFU-E cells exhibited increased percentages of EdU^+^ cells (Figure 3D), the MFI of EdU, which reflects rate of DNA synthesis during S-phase (39, 40), remained the same (Figure 3F), indicating increased DNA synthesis in *Mettl3*-deficient BFU-E and CFU-E cells without change in DNA synthesis rate. Notably, the MFI of EdU in the CFU-E^kit-lo^ cells was significantly lower compared with control CFU-E cells (Figure 3F), indicating an imbalance between the accelerated cell cycle and the reduced DNA synthesis rate, which may trigger DNA replication stress and subsequent occurrence of cell apoptosis. In contrast to BFU-E and CFU-E cells, deficiency of *Mettl3* resulted in decreased proliferation of erythroblasts, as demonstrated by the decreases in EdU^+^ erythroblasts (Figure 3E) as well as decreased EdU MFI in *Mettl3*-deficient erythroblasts (Figure 3F). Together, these findings indicate that *Mettl3* deficiency–induced defective DNA synthesis led to apoptosis of CFU-E and cell-cycle arrest of erythroblasts.

### RNA-seq analyses revealed alterations in pathways involved in DNA synthesis, DNA damage, and apoptosis in Mettl3-deficient CFU-E.

**Figure 4:**
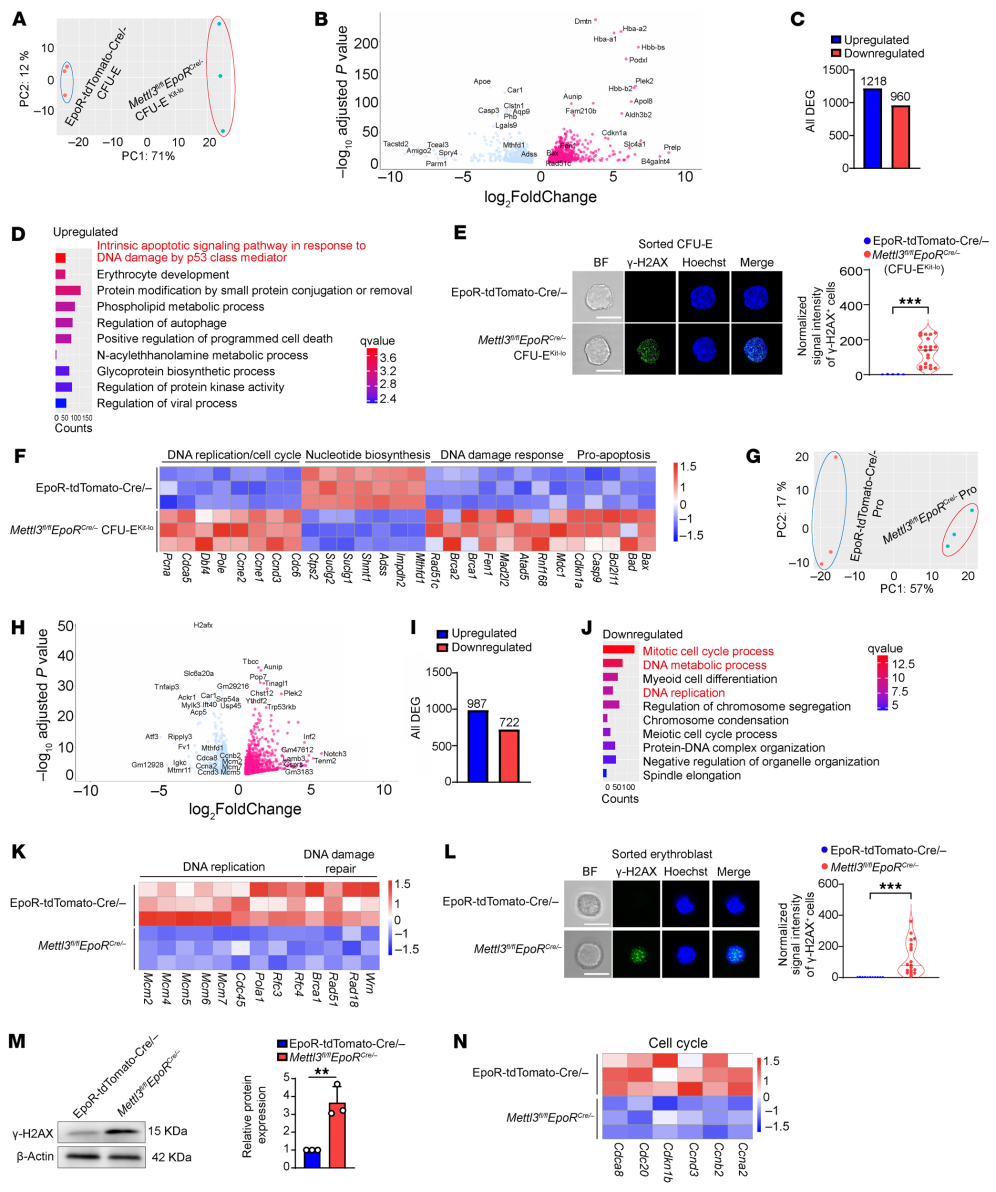
RNA-seq analysis of CFU-E cells and ProEs. (**A**) PCA of transcriptomes shows the separation between control CFU-E and *Mettl3*-deficient CFU-E^Kit-lo^ cells. (**B**) Volcano plots of differential expression genes in control CFU-E cells and *Mettl3*-deficient CFU-E^Kit-lo^ cells. (**C**) Bar plot of DEGs between BM control CFU-E and *Mettl3*-deficient CFU-E^Kit-lo^ cells. (**D**) GO enrichment analysis of genes upregulated in *Mettl3*-deficient CFU-E^Kit-lo^ cells. (**E**) Confocal images of control and *Mettl3*-deficient CFU-E^Kit-lo^ cells stained for DNA (Hoechst, blue) and γ-H2AX foci (green), and quantification of γ-H2AX immunofluorescent intensity in *Mettl3*-deficient mice CFU-E^Kit-lo^ cells, normalized to control mice CFU-E cells (*n* = 5–21/group). (**F**) Heatmap showing expression levels (TPM) of DNA replication/cell cycle, nucleotide biosynthesis, DNA damage response, and pro-apoptosis–related genes in control CFU-E cells and *Mettl3*-deficient CFU-E^Kit-lo^ cells from RNA-seq data. (**G**) PCA of transcriptomes revealing distinct clustering between BM control and *Mettl3*-deficient ProEs. (**H**) Volcano plots of DEGs in ProEs between control and *Mettl3*^fl/fl^ *EpoR*^cre/-^ mice. (**I**) Bar plot showing numbers of DEGs between BM control and *Mettl3*-deficient ProEs. (**J**) GO terms enriched for downregulated genes in *Mettl3*-deficient ProEs. (**K**) Heatmap of gene expression for genes involved in DNA replication and DNA damage repair. (**L**) γ-H2AX immunofluorescence for DNA damage in control and *Mettl3*-deficient erythroblasts (scale bar: 10 μm), and quantification of γ-H2AX immunofluorescent intensity in *Mettl3*-deficient mice, normalized to control mice erythroblast cells (*n* = 11–19/group). (**M**) Western blot analysis of γ-H2AX expression and corresponding quantification in erythroblast cells (*n* = 3/group). (**N**) Heatmap showing TPM of cell-cycle genes in control and *Mettl3*-deficient ProEs. Data are presented as the mean ± SD. Comparisons between 2 groups were performed using an unpaired 2-tailed Student’s *t* test. **P < 0.01, ***P < 0.001.

To define the molecular mechanisms for the increased apoptosis of *Mettl3*-deficient CFU-E^Kit-lo^ cells, we performed bulk RNA-seq analyses. Principal component analyses (PCA) showed clear separation of control and *Mettl3*-deficient cells (Figure 4A). We identified 2,178 differentially expressed genes (DEGs), with 1,218 upregulated and 960 downregulated in *Mettl3*-deficient CFU-E^Kit-lo^ compared with control CFU-E cells (Figure 4, B and C). The list of DEGs is provided in Supplemental Table 1. Gene Ontology (GO) analysis of the DEGs revealed that intrinsic apoptotic signaling pathway in response to DNA damage by p53 class mediator was remarkably upregulated in *Mettl3*-deficient CFU-E^Kit-lo^ cells compared with the control CFU-E cells (Figure 4D), indicating DNA damage in *Mettl3*-deficient CFU-E^Kit-lo^ cells, which was further evidenced by increased levels of phosphorylated H2AX (γ-H2AX) (Figure 4E), a marker of DNA damage and genomic instability (41, 42). Additional pathway analyses revealed that, consistent with the finding of an imbalance between the accelerated cell cycle and the reduced DNA synthesis rate in *Mettl3*-deficient CFU-E^Kit-lo^ cells, genes involved in DNA replication and the cell cycle (namely, *Cdc6*, *Ccnd3*, *Ccne1/2*, *Pole*, *Dbf4*, *Cdca5*, and *Pcna*) were upregulated, whereas genes required for nucleotide biosynthesis, including *Mthfd1*, *Impdh2*, *Adss*, *Shmt1*, *Suclg1/2*, and *Ctps2*, were downregulated in *Mettl3*-deficient CFU-E^Kit-lo^ cells compared with control CFU-E cells (Figure 4F). Figure 4F also shows upregulation of DNA damage response pathways as evidenced by the upregulated expression of persistent DNA damage markers (*Mdc1*, *Rnf168*), replication stress responders (*Atad5*, *Mad2l2*, and *Fen1*), and homologous recombination factors (*Brca1*, *Brca2*, and *Rad51c*). Finally, the expression of proapoptotic genes such as *Bax*, *Bad*, *Bcl2l11*, *Casp9*, and *Cdkn1a* was also upregulated. These findings reveal a molecular cascade whereby *Mettl3* deficiency disrupts nucleotide biosynthesis, induces replication stress and DNA damage, and ultimately triggers apoptosis in CFU-E cells.

### RNA-seq analyses revealed alterations in pathways involved in the DNA replication cell cycle in Mettl3-deficient ProEs.

To define the molecular mechanisms for the cell-cycle arrest of *Mettl3*-deficient erythroblasts, we performed bulk RNA-seq analyses on the sorted ProEs. PCA analyses showed clear separation of control and *Mettl3*-deficient ProEs (Figure 4G). A total of 1,709 DEGs were identified, with 987 upregulated and 722 downregulated in *Mettl3*-deficient ProEs compared with control ProEs (Supplemental Table 2 and Figure 4, H and I). Consistent with decreased cell proliferation of the *Mettl3*-deficient ProEs, GO enrichment analysis revealed significant downregulation of pathways related to the mitotic cell-cycle process, DNA metabolism process, and DNA replication (Figure 4J), as demonstrated by the decreased expression of key DNA replication genes such as *Mcm2*, *Mcm4-7*, *Cdc45*, *Pola1*, and *Rfc3/4*, and DNA damage repair factors such as *Brca1*, *Rad51*, *Rad18*, and *Wrn* (Figure 4K). The decreased expression of these genes strongly suggests potential DNA damage in *Mettl3*-deficient ProEs, which was confirmed by the increased γ-H2AX expression (Figure 4, L and M). Moreover, genes involved in cell-cycle regulation, such as *Ccna2*, *Ccnb2*, *Ccnd3*, *Cdkn1b*, *Cdc20*, and *Cdca8*, were also decreased in *Mettl3*-deficient ProEs (Figure 4N). Strikingly, no significant changes were detected in apoptosis-related genes in *Mettl3*-deficient ProEs, suggesting a distinct response mechanism of ProEs compared with CFU-E cells.

### Integrated m^6^A-seq and RNA-seq analysis identified Mthfd1 as a Mettl3 direct target, the downregulation of which led to defective nucleotide biosynthesis.

**Figure 5:**
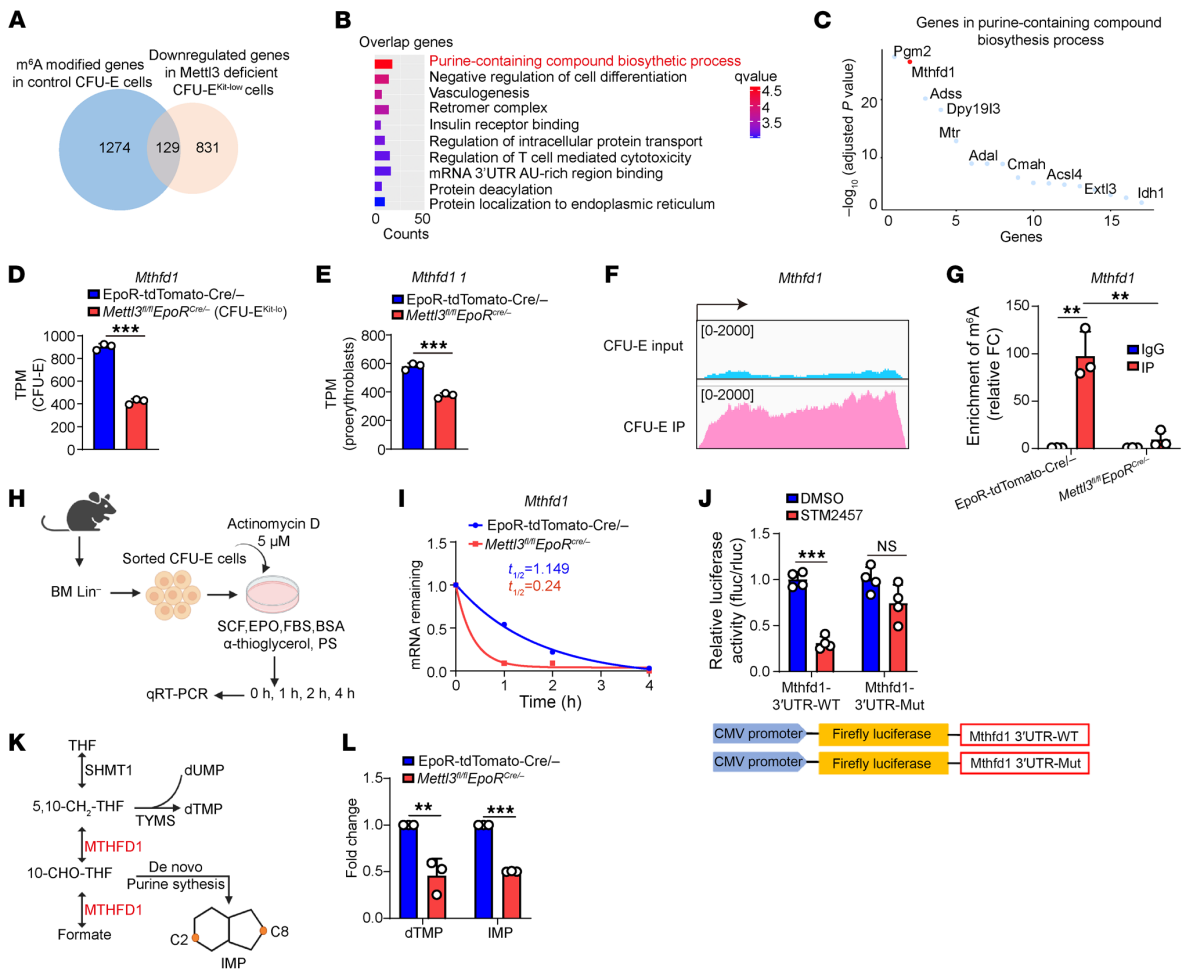
Integrated SLIM-seq/RNA-seq identified Mthfd1 as a Mettl3 target regulating erythroid nucleotide biosynthesis. (**A**) Integrated analysis of downregulated m^6^A-modified mRNAs in control and *Mettl3*-deficient CFU-E^Kit-lo^ cells. (**B**) GO enrichment analysis of the 129 overlapping genes from (A). (**C**) Significance ranking of DEGs in purine-containing compound biosynthetic processes. (**D**) Expression levels of *Mthfd1* in BM CFU-E cells from RNA-seq by TPM (*n* = 3/group). (**E**) Expression levels of *Mthfd1* in BM ProEs from RNA-seq by TPM (*n* = 3/group). (**F**) Integrative Genomics Viewer snapshots of SLIM-seq read coverage for the Mthfd1 transcriptome in control CFU-E cells, comparing Input and IP groups. (**G**) Measurement of m^6^A modification levels on Mthfd1 in control and *Mettl3*-deficient CFU-E cells by MeRIP-qPCR (*n* = 3/group). (**H**) Schematic representation of mRNA half-life measurements in actinomycin D–treated erythroid progenitor CFU-E cells. (**I**) mRNA half-life of Mthfd1 in sorted CFU-E cells, measured after actinomycin D treatment at 0, 1, 2, and 4 hours. (**J**) Dual-luciferase reporter assay identifying METTL3-regulated *Mthfd1* transcripts. The experiment used pMIR-firefly and TK–*Renilla* luciferase vectors, with WT and Mut sequences inserted at the 3′UTR of Fluc (*n* = 4/group). (**K**) Schematic diagram of MTHFD1-mediated biosynthesis pathway of dTMP and IMP. (**L**) Targeted liquid chromatography–tandem mass spectroscopy quantification of dTMP and IMP levels in erythroid cells (*n* = 3/group). Data are presented as the mean ± SD. Comparisons between 2 groups were performed using an unpaired 2-tailed Student’s *t* test. A 2-way ANOVA with Tukey’s post hoc test was used to calculate statistical significance among multiple groups. **P < 0.01, ***P < 0.001. FC, fold change; PS, penicillin-streptomycin; SCF, stem cell factor.

Next, we sought to define the upstream gene(s) that are responsible for the altered pathways in the *Mettl3*-deficient CFU-E and ProE cells. For this, we first performed a highly sensitive and efficient super-low-input m^6^A sequencing (SLIM-seq), using our previously described method (43) on the sorted CFU-E cells. A total of 1,523 genes were identified as being m^6^A modified in CFU-E cells; these are listed in Supplemental Table 3. We then integrated the m^6^A-modified mRNAs with the genes that were significantly downregulated in *Mettl3*-deficient CFU-E^Kit-lo^ cells and identified 129 overlapping genes (Figure 5A).

GO analysis of these genes revealed significant enrichment in the purine-containing compound biosynthetic process pathway (Figure 5B). Notably, the expression of methylenetetrahydrofolate dehydrogenase, cyclohydrolase, and formyltetrahydrofolate synthetase 1 (*Mthfd1*) (34, 44), the key enzyme for nucleotide biosynthesis, was the most downregulated gene in *Mettl3*-deficient CFU-E^Kit-lo^ cells (Figure 5C). Figure 5D shows that the expression of *Mthfd1* was decreased by 50%. Additionally, the expression of *Mthfd1* was also decreased in *Mettl3*-deficient ProEs (Figure 5E). Integrative Genomics Viewer (https://igv.org) showed an obvious enrichment of m^6^A in *Mthfd1* mRNA in normal CFU-E cells (Figure 5F). Additional MeRIP-qPCR experiments revealed a significant decrease in m^6^A modifications on *Mthfd1* mRNA transcripts following *Mettl3* knockout (Figure 5G).

Given that m^6^A modification regulates mRNA stability (24), we then examined stability of *Mthfd1* mRNA and found that the half-life of *Mthfd1* mRNA was decreased from 1.15 hours in control CFU-E cells to 0.24 hours in *Mettl3*-deficient CFU-E cells (Figure 5, H and I), indicating the m^6^A modification of *Mthfd1* is crucial for its stability. To further confirm that Mettl3 stabilizes *Mthfd1* mRNA in an m^6^A-dependent manner, we constructed a luciferase reporter with *Mthfd1* 3′UTR that is enriched with m^6^A modification or m^6^A-site mutant (Mut). As expected, the luciferase activity of WT, but not of Mut *Mthfd1* 3′UTR, was significantly reduced upon METTL3 inhibition, indicating that m^6^A is required for maintaining *Mthfd1* level (Figure 5J). This result indicates that METTL3 directly regulates m^6^A deposition on *Mthfd1*, thereby controlling its gene expression. As shown in Figure 5K, MTHFD1 primarily facilitates the conversion of deoxyuridine monophosphate to dTMP by supplying methyl groups to thymidylate synthase, a critical step for DNA synthesis (34). Additionally, it provides carbon units (C2 and C8) for IMP synthesis, thereby contributing to de novo purine biosynthesis (45).

We next quantified changes in metabolites using liquid chromatography–tandem mass spectrometry. Among 540 unique metabolites analyzed, dTMP and IMP were significant reduced in *Mettl3*-deficient erythroid cells compared with the control cells (Figure 5L and Supplemental Table 4). Together, these results suggest that *Mettl3*-mediated m^6^A modification is essential for the stability of *Mthfd1* mRNA, the deficiency of which led to defective nucleotide biosynthesis, which, in turn, induced DNA replication stress, leading to DNA damage.

### METTL3 inhibition impaired human erythropoiesis via similar mechanisms as in mice.

**Figure 6:**
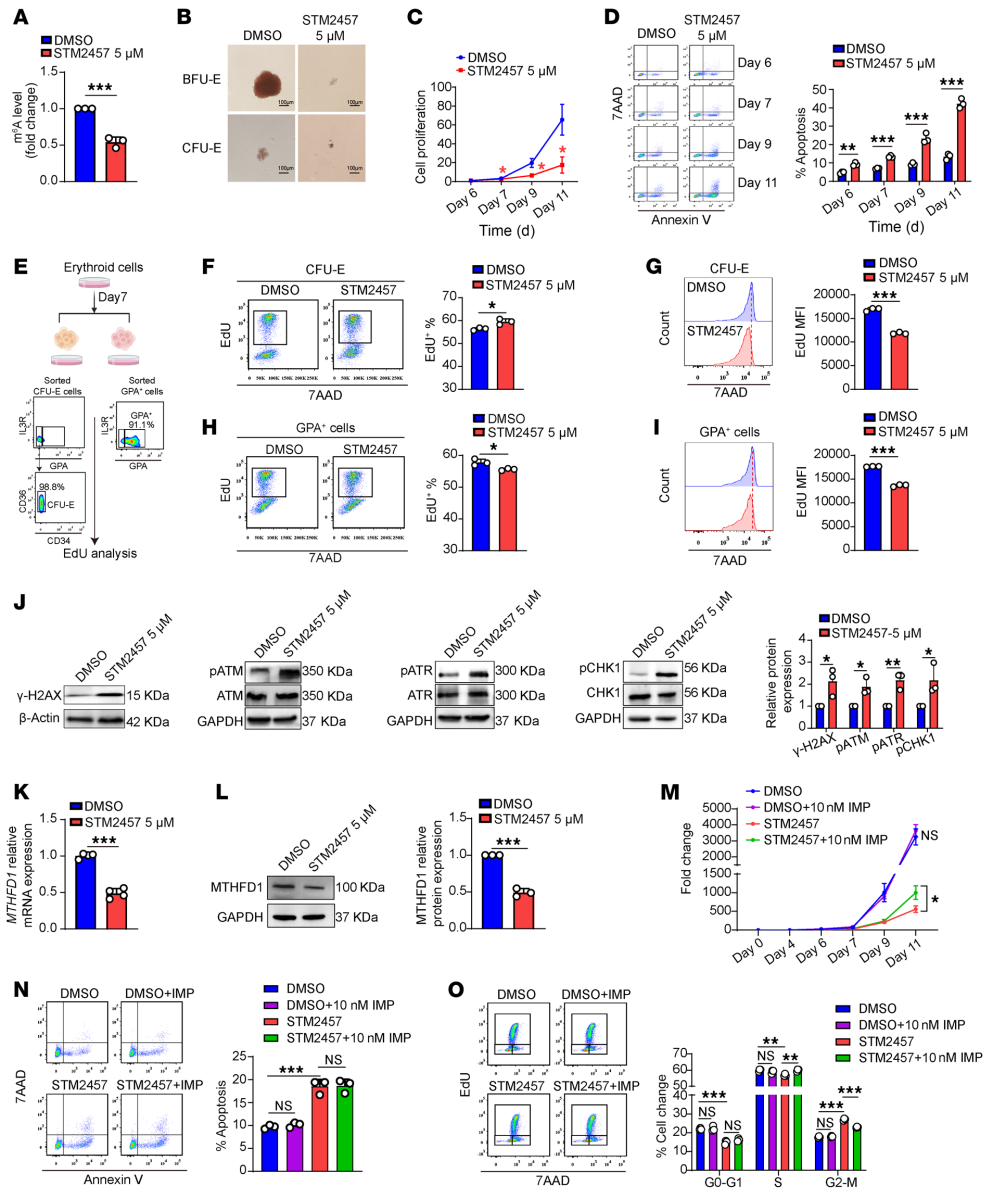
METTL3 inhibition impaired human erythropoiesis. (**A**) Global m^6^A levels in total RNA after DMSO or STM2457 treatment (*n* = 3/group). (**B**) Colony-forming ability of progenitor cells on day 6. Scale bar: 100 μm. (**C**) Proliferation curve of human CD34^+^ cells treated with DMSO or STM2457 (*n* = 3/group). (**D**) Representative flow cytometry profiles and quantification of erythroid cell apoptosis (*n* = 3/group). (**E**) Schematic illustration and purity assessment of sorted human CFU-E and GPA^+^ cells for EdU assay. (**F**) Flow cytometry quantified the proportion of EdU^+^ erythroid cells in sorted human CFU-E cells and (**G**) quantification of the EdU MFI in CFU-E cells during S phase (*n* = 3/group). (**H**) The proportion of EdU^+^ erythroid cells was determined by flow cytometry in sorted human GPA^+^ cells. (**I**) Measurement of EdU MFI in GPA^+^ cells during S phase (*n* = 3/group). (**J**) Representative Western blot analysis of γ-H2AX, pATM, pATR, and pCHK1 in erythroid cells treated with DMSO or STM2457 on day 7 (*n* = 3/group). (**K**) qRT-PCR measurement of *MTHFD1* mRNA levels after METTL3 inhibition (*n* = 3/group). (**L**) Western blot analysis of MTHFD1 protein level in METTL3-deficient cells (*n* = 3/group). (**M**) Exogenous IMP supplementation partially rescued the proliferation deficit in STM2457-treated erythroid cells (*n* = 3/group). (**N**) Analysis of the effect of exogenous IMP on apoptosis in STM2457-treated erythroid cells (*n* = 3/group). (**O**) Exogenous IMP supplementation partially rescued the cell-cycle defect in STM2457-treated erythroid cells (*n* = 3/group). Data are presented as mean ± SD. Comparisons between 2 groups were performed using an unpaired 2-tailed Student’s *t* test. A 2-way ANOVA with Tukey’s post hoc test was used to calculate statistical significance among multiple groups. *P < 0.05, **P < 0.01, ***P < 0.001.

A previous study implicated the role of METTL3 in human erythropoiesis in vitro (32), but the underlying mechanisms remain incompletely understood. To examine whether similar mechanisms exist between murine and human, we inhibited METTL3 activity using STM2457, a highly selective inhibitor of METTL3 (46). As expected, STM2457 treatment led to significant reduction at day 7 of global m^6^A levels in erythroid cells cultured in vitro (Figure 6A). Erythroid colony assays revealed that STM2457 treatment impaired the colony-forming ability of erythroid progenitors, as demonstrated by the dramatic decreases in colony sizes of BFU-E and CFU-E cells (Figure 6B). STM2457 treatment also inhibited the growth of erythroblasts (Figure 6C). Flow cytometry analysis of apoptosis, using annexin V and 7AAD staining, showed STM2457 treatment led to increased apoptosis of erythroid progenitors as well as erythroblasts (Figure 6D). In alignment with in vivo findings, cell-cycle analysis (Figure 6E) revealed differential effects of STM2457 on CFU-E cells and erythroblasts. Although STM2457 treatment led to the increases of EdU^+^ CFU-E cells (Figure 6F) along with a decreased DNA synthesis rate (Figure 6G), it led to decreases in both the percentage of EdU^+^ erythroblasts and DNA synthesis rate (Figure 6, H and I).

At the molecular level, inhibition of METLL3 in CFU-E cells led to upregulation of pro-apoptotic genes and genes involved in DNA damage response, as well as upregulation of genes involved in cell-cycle regulators and DNA replication (Supplemental Figure 9, A–D). A different expression pattern was observed in METTL3-inhibited erythroblasts, which also showed increased pro-apoptotic and DNA damage response signaling, concurrent with decreased expression of cell cycle and DNA replication (Supplemental Figure 9, E–H) genes. Additionally, shRNA-mediated knockdown of *METTL3* also led to reduction in protein level (Supplemental Figure 10A) and global m^6^A levels (Supplemental Figure 10B), impairment of erythroid colony formation (Supplemental Figure 10C), inhibition of erythroblast growth (Supplemental Figure 10D), and increase in apoptosis of erythroid progenitors and erythroblasts (Supplemental Figure 10E). Notably, both STM2457 treatment and shRNA-mediated *METTL3* knockdown resulted in increased expression of γ-H2AX and activated DNA damage sensors, such as pATM, pATR, and pCHK1 (Figure 6J and Supplemental Figure 10F), indicating increased DNA damage. Importantly, METTL3 inhibition also led to decreased expression of MTHFD1 in mRNA (Figure 6K) and protein (Figure 6L and Supplemental Figure 10G) levels. In addition to the downregulation of MTHFD1, STM2457 treatment led to decreased expression of heme synthesis and HGB-related genes (*ALAS2*, *FECH*, and *HBA*). These effects mirrored the in vivo findings and were accompanied by a decrease in the typically high levels of *HBG* in vitro. Unexpectedly, *HBB* expression was increased by STM2457 treatment (Supplemental Figure 11, A–E).

Having shown that deletion of *Mettl3* in mice led to decreased levels of dTMP and IMP, we performed a rescue assay by supplementing with thymidine (the precursor molecule of dTMP) and IMP in a human erythroid culture system. The results demonstrated that whereas thymidine supplementation failed to restore erythroid cell proliferation (Supplemental Figure 12A), IMP supplementation partially rescued the erythroid defects (Figure 6M). Although IMP did not mitigate erythroid cell apoptosis (Figure 6N), it effectively restored the cell-cycle progression (Figure 6O). These findings indicate that the reduction in IMP contributes partially to the erythroid defects induced by METTL3 inhibition. Consistent with this, intraperitoneal injection of IMP in Mettl3-deficient mice led to partial recovery of RBC count, along with a trending elevation in HCT levels (Supplemental Figure 12B). These findings indicate that deficiency of METTL3 impairs human erythropoiesis via similar mechanisms as in mice.

### Knockdown of MTHFD1 in human erythroid cells mimicked the defects caused by METTL3 inhibition.

**Figure 7:**
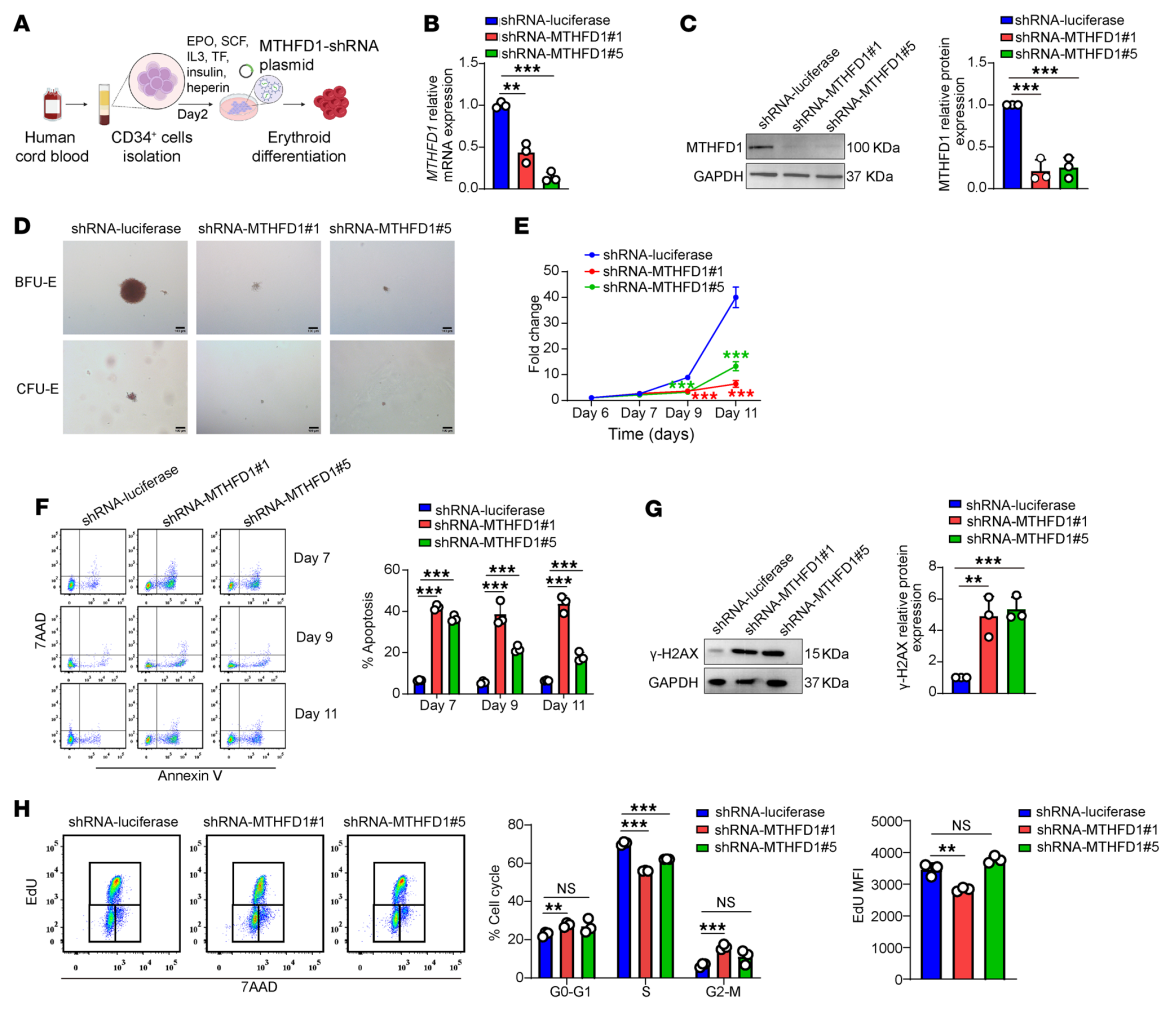
*MTHFD1* deficiency in human erythroid cells phenocopied METTL3 inhibition defects. (**A**) Schematic of lentiviral *MTHFD1*-shRNA transduction and erythroid differentiation induction (*n* = 3/group). (**B**) qRT-PCR analysis of *MTHFD1* knockdown efficiency at mRNA level in shRNA-luciferase, 2 groups of shRNA-*MTHFD1* transduced cells (labeled shRNA-*MTHFD1*#1, and shRNA-*MTHFD1*#5 in the figure) (*n* = 3/group). (**C**) Western blot analysis of MTHFD1 knockdown efficiency and corresponding quantification at the protein level (*n* = 3/group). (**D**) Colony-forming ability of luciferase-control and *MTHFD1*-knockdown cells at day 6. Scale bar: 100 μm. (**E**) Growth curves of luciferase-control and *MTHFD1*-knockdown cells (*n* = 3/group). (**F**) Flow cytometric analysis of apoptosis by 7AAD/annexin V staining at days 7, 9, and 11 (*n* = 3/group). (**G**) Western blot analysis of γ-H2AX and corresponding quantification in luciferase-control and *MTHFD1*-knockdown cells at day 7 (*n* = 3/group). (**H**) The proportion of EdU^+^ cells was quantified by flow cytometry in luciferase-control and *MTHFD1*-knockdown cells on day 7 and quantification of MFI in EdU^+^ cells (*n* = 3/group). Data are presented as the mean ± SD. Two-way ANOVA with Tukey’s post hoc test was used to calculate statistical significance among multiple groups. **P < 0.01, ***P < 0.001.

To gain further support that deficiency of *METTL3* led to impaired human erythropoiesis via downregulation of MTHFD1, we examined the effects of *MTHFD1* knockdown on human erythropoiesis (Figure 7A). Efficient knockdown of *MTHFD1* was achieved at both mRNA (Figure 7B) and protein (Figure 7C) levels. Notably, *MTHFD1* knockdown phenocopied the effects of *METTL3* knockdown on human erythropoiesis, as demonstrated by decreases in BFU-E and CFU-E colony formation (Figure 7D), inhibition of erythroid progenitor and erythroblast proliferation (Figure 7E), and increased apoptosis of both erythroid progenitors and erythroblasts (Figure 7F). Moreover, *MTHFD1* knockdown also led to increased DNA damage, as evidenced by an increase in γ-H2AX level (Figure 7G). Cell-cycle analysis revealed that *MTHFD1* deficiency inhibited the erythroid cell cycle and slowed the DNA synthesis rate (Figure 7H). These findings further support that METTL3 regulates erythropoiesis, at least in part, via downregulation of MTHFD1.

## Discussion

In this study, we investigated the functional role of Mettl3-mediated m^6^A modification of mRNA in erythropoiesis in vivo and its underlying cellular and molecular mechanisms. We showed that deletion of *Mettl3* in erythroid cells led to microcytic hypochromic anemia. At the cellular level, *Mettl3* deletion led to apoptosis of CFU-E cells and cell-cycle arrest of erythroblasts due to DNA damage. At the molecular level, we identified Mthfd1 as a direct target of Mettl3. Importantly, deletion of *Mettl3* led to the decreased expression of Mthfd1 due to instability of the *Mthfd1* mRNA along with decreased levels of dTMP and IMP, which triggers DNA replication stress, leading to DNA damage.

We crossed *EpoR-tdTomato-Cre* and *Mettl3^fl/fl^* mice to conditionally delete *Mettl3* expression. Notably, whereas *Mettl3^fl/fl^ EpoR^Cre/Cre^* mice were perinatal lethal, *Mettl3^fl/fl^ EpoR^Cre/–^* mice were viable along with anemia. Given that EpoR was expressed in HSCs (33), that *EpoR^Cre/–^* led to an approximately 50% reduction of *Mettl3* in HSPCs, and that *Mettl3^fl/fl^ Vav^Cre/–^* mice in which *Mettl3* was completely deleted in fetal liver HSPCs were perinatal lethal (28), we speculate that the perinatal lethality of the *Mettl3^fl/fl^ EpoR^Cre/Cre^* mice is associated with more efficient deletion of *Mettl3* in HSCs. Furthermore, our findings that *Mettl3^fl/fl^ EpoR^Cre/–^* mice did not exhibit significant changes in HSPCs and that deletion of *Mettl3* in EBI macrophages did not affect erythropoiesis strongly suggest that anemia of *Mettl3^fl/fl^ EpoR^Cre/–^* mice is primarily attributed to the complete deletion of *Mettl3* in erythroid cells.

An interesting finding of our study is the microcytic and hypochromic nature of anemia of the *Mettl3^fl/fl^ EpoR^Cre/–^* mice along with the decreased expression of *Alas2*, *Fech*, *Hba*, and *Hbb* genes. We also found that high levels of m^6^A were detected on *Alas2*, *Fech*, *Hba*, and *Hbb*, and that Mettl3 deletion led to reduction of m^6^A levels on *Fech*, *Hba*, and *Hbb*, but not *Alas2*. Our findings are in line with a previous finding that *Hba* and *Hbb* mRNAs were highly enriched m^6^A modification (43). Together, these findings demonstrate the role of Mettl3 in HGB production and may have implications in understanding the mechanisms for microcytic hypochromic anemia in vivo. Interestingly, inhibition of METTL3 in cultured erythroblasts in vitro led to the downregulation of *FECH*, *ALAS2*, *HBA*, and *HBG* but induced upregulation of *HBB*. This pattern was consistent with the previous finding that loss of METTL3 in HEL-GYPA–positive cells affected these genes (32). Future studies will focus on elucidating the molecular mechanisms underlying the differential responsiveness of *HBB* to METTL3 depletion in vivo versus in vitro.

In analyzing the erythroid progenitors by flow cytometry using Kit as one of the surface markers, we noted a significant reduction in Kit expression on CFU-E cells, with a subset of CFU-E cells (CFU-E^kit-lo^) exhibiting drastically decreased Kit expression levels. In parallel with decreased surface expression of Kit, the mRNA levels of Kit were also decreased, indicating the decreased expression of Kit originating at the transcriptional level. Interestingly, m^6^A-seq of CFU-E cells did not reveal significant m^6^A modification enrichment on Kit mRNA, suggesting that *Mettl3* deficiency-induced Kit downregulation occurred independently of m^6^A modification. Given that EPO has been reported to downregulate Kit expression in leukemic ProEs in vitro (37, 47), and that EPO levels were increased in *Mettl3^fl/fl^ EpoR^Cre/–^* mice, we hypothesized that the increased EPO levels contribute to downregulation of Kit in the CFU-E. To test this, we examined the expression of Kit on erythroid progenitors from EPO-injected mice and found that, indeed, EPO injection led to decreased expression of Kit on CFU-E cells in vivo. However, although the extent of decreased expression on *Mettl3*-deficient CFU-E cells could be explained by the increased EPO levels, the mechanisms for the drastic decrease of Kit expression in *Mettl3*-deficient CFU-E^kit-lo^ cells have yet to be defined.

Erythropoiesis is characterized by rapid cell division during which genome integrity needs to be maintained. However, the mechanisms for the maintenance of genome stability in erythroid cells remain largely unclear. One important finding of the present study was that deletion of *Mettl3* in erythroid cells induced DNA damage, demonstrating the critical role of Mettl3-mediated m^6^A modification in maintaining the genome stability of erythroid cells. It is interesting to note that although *Mettl3* deficiency induced DNA damage in both CFU-E cells and erythroblasts, it elicited distinct cellular responses: apoptosis of CFU-E cells and cell-cycle arrest of erythroblasts, demonstrating the stage-specific role of Mettl3. Although in vitro METTL3 inhibition faithfully reproduces the CFU-E apoptosis seen in vivo, it induces both cell-cycle arrest and apoptosis in erythroblasts. This differential response of erythroblasts to *Mettl3* depletion in vivo versus in vitro may be explained by two mechanisms. On the one hand, it could be attributed to the sustained upregulation of the anti-apoptotic factor Bcl-XL during the early stages of terminal erythroid differentiation in vivo, whereas the in vitro system only upregulates Bcl-XL at late stages (48). On the other hand, the anemia resulting from *Mettl3* knockout may create a critical compensatory demand for erythrocytes in vivo, thereby triggering a negative feedback regulatory mechanism.

A previous study reported that knockdown of *METTL3* in human erythroleukemia cells resulted in downregulation of pathway involved in the purine nucleoside metabolic pathway (32). However, the direct METTL3 target genes in this pathway were not defined. Here, using integrated m^6^A-seq and RNA-seq along with biochemical studies, we have identified Mthfd1, a key enzyme involved in nucleotide biosynthesis (34), as a direct target of Mettl3. Although IMP supplementation significantly improved erythroid proliferation, it did not achieve a complete rescue, suggesting METTL3 regulates erythropoiesis through additional targets beyond MTHFD1. Our preliminary data indicate that within the same purine-containing compound biosynthetic pathway, adenylosuccinate synthase (ADSS) and phosphoglucomutase 2 (PGM2) may also be potential target genes of METTL3. During nucleotide synthesis, ADSS, downstream of MTHFD1, primarily regulates the conversion of IMP to AMP (49), whereas PGM2 is mainly involved in nucleotide sugar synthesis (50). We hypothesize that METTL3 may regulate erythropoiesis by mediating multiple steps of nucleotide metabolism. Further investigation is needed to test our hypothesis. This view aligns with a recent study that found Mettl16, a member of the RNA m^6^A methyltransferase family, plays a critical role in fetal liver erythropoiesis by safeguarding genome integrity of erythroblasts via modulating m^6^A deposition on transcripts of DNA repair–related genes (51). Together, these findings demonstrate the essential role of m^6^A modification in the maintenance of genome stability in erythroid cells.

In summary, we have established a critical functional role of Mettl3-mediated m^6^A modification in erythropoiesis in vivo and have identified the underlying molecular mechanisms. Importantly, METTL3 plays similar role in human erythropoiesis via similar mechanisms. Given that altered m^6^A modification is associated with various human diseases (52), our study highlights the need for studies to systematically examine m^6^A modification patterns in erythroid disorders and elucidate the contributions of METTL3 to disease pathogenesis, which may open potential therapeutic avenues.

## Methods

### Sex as a biological variable.

Our study examined male and female animals, and similar findings are reported for both sexes.

### Mice.

The mice used in this study were on a C57BL/6 background. The *Mettl3^fl/fl^* mice were provided by Pengyuan Yang at the Institute of Biophysics, Chinese Academy of Sciences, and the *EpoR-tdTomato-Cre* (33) and *CD169-tdTomato-Cre* mice were generated and characterized in our laboratory. The mice were raised in a safe facility. Male and female mice, 10–16 weeks old, were used for experiment. All mice were bred and maintained at Zhengzhou University.

### Measurement of total m^6^A levels in cells.

Total RNA was isolated from cells using an RNA isolation kit (Vazyme, RC112-01). We analyzed 200 ng total RNA, using the m^6^A RNA Methylation Quantification Kit (Abcam, ab185912) according to the manufacturer’s instructions.

### Measurement of blood parameters.

Blood parameters of the mice were measured using an automated hematology analyzer (ADVIA 2120i).

### Measurement of serum EPO levels by ELISA.

Blood samples were collected and allowed to stand at room temperature for 2 hours, after which they were subjected to centrifugation at 300*g* for 5 minutes. The serum requires a 2-fold dilution, which was carried out in accordance with the manufacturer’s instructions (R&D system, MEP00B).

### Mouse erythroid progenitor colony-forming assay.

BFU-E cells were plated in 35 mm dishes containing 1.1 mL MethoCult M3434 medium (Stem Cell Technologies, 03434), and colonies were counted on day 7. CFU-E cells were cultured in MethoCult M3334 medium (Stem Cell Technologies, 03334), with colony counts performed at 48 hours using an inverted microscope (Olympus).

### Preparation of Lineage^–^ cells.

BM cells were isolated from mouse tibiae and femurs using PBS containing 2% FBS and 2 mM EDTA (buffer 1). Single-cell suspensions were obtained by filtering solutions through 70 μm cell strainers. Total BM cells were first incubated with CD16/32 Fc-blocking antibody (eBioscience, 553142). Next, the cells were stained with a cocktail of biotinylated lineage markers that included Biotin-Gr-1 (eBioscience, 13-5931-75), Biotin-CD11b (eBioscience, 13-0112-75), Biotin-CD3e (eBioscience, 13-0031-75), Biotin-B220 (eBioscience, 103204), and Biotin-Ter119 (eBioscience, 13-5921-75) and incubated at 4°C for 30 minutes. Subsequently, the cells were incubated with anti-biotin microbeads (Miltenyi Biotec, 130-090-485) at 4°C for 15 minutes, followed by magnetic enrichment.

### Surface marker staining and flow cytometry of erythroid progenitors.

Lineage^–^ cells (hereafter, Lin^–^) were stained with PerCP-streptavidin and the following fluorescently labeled antibodies: BV421-anti-CD16/32 (BioLegend, 101331), eF450-anti-CD41(Invitrogen, Thermo Fisher Scientific, 48-0411-80), APC-anti-CD34 (BioLegend, 119310), APC-anti-Sca1 (BioLegend, 108112), APC-Cy7-anti-CD117 (BioLegend, 105826), and BV750-anti-CD71 (BD, 747095). Erythroid progenitors were defined as BFU-E: 7AAD^–^Lin^–^CD16/32^–^CD41^–^CD34^–^Sca1^–^Kit^+^CD71^–^, CFU-E: 7AAD^–^Lin^–^CD16/32^–^CD41^–^CD34^–^Sca1^–^Kit^+^CD71^+^. All staining steps were performed at room temperature for 30 minutes in the dark. After incubation, cells were centrifuged at 300*g* for 5 minutes, resuspended in 200 μL of buffer 1, and stained with the viability marker 7AAD (0.5 μg/mL) for 10 minutes in the dark. Finally, cells were transferred to flow cytometry tubes and analyzed using a BD LSRFortessa flow cytometer.

### Surface marker staining and flow cytometry of erythroblasts.

A single-cell suspension of mouse BM containing 1 × 10^6^ cells was washed once with 1 mL of buffer 1 and centrifuged at 300*g* for 5 minutes. After removing the supernatant, cells were blocked with CD16/32 Fc-blocking antibody (eBioscience, 553142) for 10 minutes at 4°C. The following antibodies were then added for staining: APC-Cy7–conjugated anti–mouse CD45 (BD, 557659), APC-Cy7-conjugated anti–mouse CD11b (BD, 557657), APC-Cy7–conjugated anti–mouse Gr-1 (BD, 557661), BV421-conjugated anti–mouse Ter119 (BD, 560504), and APC-conjugated anti-mouse CD44 (BD, 559250). Cells were incubated with the antibody on ice for 30 minutes in the dark, followed by a wash with 1 mL buffer 1 and centrifugation at 300*g* for 5 minutes. The pellet was resuspended in 200 μL buffer 1 and stained with the viability marker 7AAD (0.5 μg/mL) for 10 minutes in the dark. Finally, the cells were transferred to flow cytometry tubes and analyzed using a BD LSRFortessa flow cytometer.

### Sorting erythroid progenitors and erythroblasts.

The Lin^–^ cells were suspended and stained with multiple fluorescence-labeled antibodies targeting erythroid progenitors. For erythroblast cells, total BM single cells were stained with fluorescence-labeled erythroid-specific antibodies. Cell sorting was performed using a BD FACSymphony S6 flow cytometer.

### Apoptosis analysis in vivo.

Cells were stained with erythroid cell–specific antibodies, then washed with 1 mL of PBS, and centrifuged at 300*g* for 5 minutes at 4°C. After 2 washes, the cell pellet was resuspended in 200 μL binding buffer (KeyGEN BioTECH, KGA1102-100). FITC-conjugated annexin V (2 μL) was added, followed by incubation at room temperature in the dark for 10 minutes. Subsequently, 2 μL of 7AAD was added, and samples were analyzed after using a BD LSRFortessa flow cytometer.

### EdU incorporation assay in vivo.

The EdU incorporation assay was performed using the Click-iT Plus EdU Flow Cytometry Assay Kit (Invitrogen, Thermo Fisher Scientific, C10645). Sterile-packaged EdU powder was dissolved in 1× PBS to a concentration of 2.5 mg/mL, and EdU was administered via intraperitoneal injection at a dose of 0.5 mg per mouse. After 30 minutes, the mice femurs and tibias were harvested. BM cells were isolated and washed to obtain a single-cell suspension. Erythroid progenitor cells and erythroid cells were labeled with surface markers using flow cytometry antibodies, as previously described in *Surface marker staining and flow cytometry of erythroblasts*. After 30 minutes of incubation, the cells were washed twice with 3 mL of PBS/1% BSA. The supernatant was removed, and 100 μL Click-iT Fixative (Component D) was added to the centrifuge tube, followed by thorough mixing. The cells were then incubated for 15 minutes at room temperature in the dark. After 2 additional washes with PBS/1% BSA, the supernatant was discarded, and 100 μL 1× Perm/Wash Buffer was added. The cells were vortexed and incubated for 15 minutes. The Click-iT reaction mix was prepared as follows: 438 μL PBS, 10 μL Copper Protectant (Component F), 2.5 μL Sodium Azide Working Solution (containing Alexa Fluor 350 Picolyl Azide [Component B]), and 50 μL of 1× Click-iT EdU Buffer Additive (Component G). The Click-iT reaction mixture (500 μL total) was added to each sample, vortexed, and mixed thoroughly. The samples were incubated for 30 minutes at room temperature in the dark. Following incubation, the cells were washed once with 1× PBS/1% BSA and the supernatant was removed. The cell pellet was resuspended in 200 μL 1× PBS/1% BSA, and the cells were transferred to a flow cytometry tube. Samples were analyzed using a BD FACSymphony S6 flow cytometer.

### Cytospin and Giemsa staining.

The cells were resuspended in 200 μL buffer 1 and subjected to cytospin using a Thermo Scientific Shandon 4. Giemsa staining was performed using 1 mL of Giemsa concentrate (Solarbio, G1015) mixed with 9 mL Giemsa Diluent (Solarbio, G1010) and thoroughly blending. The cytospin slides were fixed in methanol for 3 minutes. After air drying, a few drops of the diluted Giemsa staining solution were applied to cover all cells, followed by incubation for 15 minutes at room temperature. After staining, the slide was rinsed gently with tap water, avoiding direct flow onto the cells or spillage of the staining solution. After drying, the slides were observed and imaged with an Olympus microscope.

### Immunofluorescence staining.

Immunofluorescence staining was performed on sorted erythroid progenitor cells that were initially suspended in 1.5 mL buffer 1 and centrifuged at 300*g* for 5 minutes at 4°C to collect the cell pellet. The cells were fixed with 4% paraformaldehyde for 15 minutes at room temperature, followed by permeabilization in PBS containing 5% BSA and 0.2% Triton X-100 for 10 minutes. After blocking for 1 hour, the cells were incubated overnight at 4°C with γ-H2AX primary antibody (Abcam, ab81299) diluted at 1:250 in blocking buffer. After thorough washing, the cells were incubated with secondary antibodies (ABclonal, AS011) at a 1:100 dilution for 1 hour at room temperature, then counterstained with Hoechst 33342 for nuclear visualization. The stained cells were seeded onto Thermo Scientific Nunc Lab-Tek II chamber slides (155382) and imaged using a Zeiss LSM780 confocal laser scanning microscope equipped with a ×100 oil immersion objective (Carl Zeiss Microscopy). For imaging, an appropriate volume of cell suspension was added to the culture dish, and images were acquired and processed using ZEN software.

### Measurement of mRNA half-life.

CFU-E cells were isolated from mice and cultured in IMDM medium supplemented with 0.2 U/mL human EPO, 10 ng/mL murine stem cell factor, 30% FBS, 1% BSA, 0.1 mM α-thioglycerol, and 1% penicillin-streptomycin. Cells were then treated with 5 μg/mL actinomycin D to block transcription. At designated time points (0, 1, 2, and 4 hours), cells were harvested for RNA extraction using the RNA isolation kit. mRNA levels were quantified by qRT-PCR.

The mRNA decay kinetics were determined by establishing that the rate of mRNA concentration decrease over time (dC/dt) is proportional to both the decay rate constant (Kdecay) and current mRNA concentration (C), as expressed by the following equation: dC/dt = –Kdecay × C. The degradation rate constant was calculated using the logarithmic relationship ln(C/C0) = –Kdecay × *t*, where C0 represents initial mRNA concentration. To derive the mRNA half-life (*t_1/2_*), we solved for the time required for mRNA levels to decrease to 50% of their initial value (C/C0 = 0.5). Substituting into the equation yielded ln(0.5) = –Kdecay × *t_1/2_*, which simplifies to the fundamental half-life equation *t_1/2_* = ln (2)/Kdecay.

### Luciferase reporter assay.

HEK293T cells were cultured in 24-well plates until reaching 70%–80% confluence (approximately 24 hours). Cells were cotransfected with 100 ng reporter plasmids (either pMIR-Mthfd1-3′UTR-WT or pMIR-Mthfd1-3′UTR-Mut) and 20 ng *Renilla* luciferase control plasmid (pRL-TK) using HiGene transfection reagent (POWFLY, C1506). After transfection, cells were treated with either DMSO (vehicle control) or the METTL3 inhibitor STM2457 (Sellect, S9870) (5 μM) for at least 24 hours. Luciferase activities were then measured using the Dual-Luciferase Reporter Assay System (Promega, E1910) according to the manufacturer’s instructions, with firefly luciferase (Fluc) signals normalized to *Renilla* luciferase values for data analysis.

### MeRIP-qPCR.

Protein A beads (Life Technologies, 10001D) were thoroughly resuspended by vortexing for 30 seconds. For each sample, 10 μL beads was transferred and combined with 1 mL IP buffer without RNase inhibitor. After gentle mixing by pipetting or vortexing, the beads were allowed to settle, followed by supernatant removal. The washed beads were then resuspended in 200 μL IP buffer. This washing step was repeated 3 times. Bead-antibody incubation was performed by combining the beads with 0.5 μg rabbit anti-m^6^A monoclonal antibody (Abcam, ab151230), 0.5 μg rabbit IgG antibody (Proteintech, 30000-0-AP), and 5 μL 100 μM short primer, followed by incubation at 4°C for 2 hours with rotation. Upon completion of incubation, the supernatant was discarded, and the beads-antibody complexes were subjected to 3 additional washes with 1 mL IP buffer each. The washed complexes were finally resuspended in 200 μL IP buffer containing RNase inhibitor (0.4 U/μL) (Thermo Fisher Scientific, EO0384).

For RNA sample preparation, total RNA was first extracted, then heated at 65°C for 5 minutes and immediately cooled on ice. The total RNA was equally divided into 3 tubes designated as Input, IgG, and IP groups. RNA IP was performed by adding the IgG and IP group RNA to the beads-antibody complexes and incubating for another 2 hours at 4°C on a rotator. A sequential washing procedure was carried out by adding 200 μL of each specified buffer, gently mixing, allowing the beads to settle, and carefully removing the supernatant. The sequential washing procedure was as follows: 2 washes with IP buffer, 2 with low-salt buffer, 2 with high-salt buffer, and 1 final wash with IP buffer. Elution was achieved by adding 10 μL diethyl pyrocarbonate–treated water to the beads, heating at 94°C for 2 minutes, and collecting the supernatant as the eluate. The eluted RNA was subsequently used for reverse transcription and qRT-PCR analysis.

### RNA isolation, cDNA synthesis, and qRT-PCR.

Total RNA was extracted from erythroid progenitor cells using an RNA isolation kit (Vazyme, RC112-01). The extracted RNA was reverse transcribed into cDNA using the EcoDry Premix (Oligo dT) Kit (TaKaRa, 639543). Subsequent qRT-PCR analysis was conducted with 2× ChamQ Universal SYBR qPCR Master Mix (Vazyme, Q711-02) on a Roche Real-Time PCR System. All the primer sequences are listed in Supplemental Table 5.

### In vitro human erythroid culture.

Human cord blood–derived CD34^+^ cells were isolated, expanded, and differentiated into erythroid lineage. CD34^+^ cells were isolated using anti-CD34 magnetic beads (MiltenyiBiotec, 130046703). Erythroid differentiation was induced in a 3-phase culture system, as follows: The day of CD34^+^ cell harvest was designated as day 0. The basic culture medium consisted of IMDM supplemented with 2% human peripheral blood plasma, 3% human A/B serum, 200 μg/mL human holo-transferrin, 3 IU/mL heparin, and 10 μg/mL insulin. During the first phase (days 0–7), CD34^+^ cells were cultured in the presence of 10 ng/mL stem cell factor, 1 ng/mL IL-3, and 3 IU/mL EPO. In the second phase (days 7–11), IL-3 was omitted from the culture medium. In the third phase (days 11–15), cells were maintained in the base medium supplemented with 1 IU/mL EPO and 1 mg/mL transferrin.

### Human erythroid progenitor cell colony-forming assay.

A total of 200 erythroid cells were harvested on day 6 and suspended in IMDM supplemented with 2% FBS and 1% penicillin-streptomycin. The cells were then cultured in 35 mm dishes containing 1.1 mL of MethoCult H4434 Classic Medium (Stem Cell Technologies, 04434) for BFU-E assays or EPO-only MethoCult H4330 medium (Stem Cell Technologies, 04330) for CFU-E assays. Cultures were maintained at 37°C with 5% CO_2_, and colonies were counted on days 7 and 14.

### RNA-seq.

Total RNA was extracted using TRIzol reagent. RNA-seq libraries were prepared from 1 ng total RNA using the Smart-seq2 kit (TaKaRa, 634470) according to the manufacturer’s protocol. First-strand cDNA was synthesized with Oligo-dT primers, followed by PCR amplification and magnetic bead purification (Beckman, A63881). Library quality inspection was conducted using the PerkinElmer LabChip GX Touch and StepOnePlus Real-Time PCR System. Qualified libraries were sequenced on the Illumina Hiseq platform for PE150 sequencing. For RNA-seq data analysis, raw sequencing data were filtered by fastp (0.23.4), and transcript-level expression was quantified using Kallisto (version 0.48.0) with the mouse reference genome (mm10), and then merged to the gene level by the R package tximport. Differential expression analysis was performed by DEseq2 (version 2_1.44.0) for pairwise comparisons, with the cutoff of a fold change of 1.5 or greater, an adjusted *P* of <0.05, and a minimum of 1 transcript per million (TPM) for at least in 1 group. PCA was done on log-transformed normalized counts in gene expression analysis. GO enrichment analyses were applied by Metascape. GO terms and pathways with a *q* value of less than 0.001 were considered significant.

### SLIM-seq.

Total RNA was isolated from CFU-E erythroid progenitor cells using TRIzol reagent. For m^6^A IP, 0.5 μg of anti-m^6^A antibody (Abcam, ab151320) was pre-bound to Protein A magnetic beads (Thermo Fisher Scientific, 80103G) in IP buffer (10 mM Tris-HCl pH 7.4, 100 mM NaCl, 0.1% NP-40, 0.4 U/μL RNasin) with rotation at 4°C for 2 hours. Subsequently, 100 ng of RNA was added to each sample and incubated at 4°C for an additional 2 hours. The beads were washed twice with 200 μL IP buffer, twice with low-salt buffer (10 mM Tris-HCl pH 7.4, 50 mM NaCl), twice with high-salt buffer (10 mM Tris-HCl pH 7.4, 500 mM NaCl, 0.1% NP-40, 0.4 U/μL RNasin), and once with IP buffer. Captured RNA was eluted by incubating beads at 94°C for 2 minutes in 10 μL diethyl pyrocarbonate–treated water.

For library preparation, both input RNA and IP RNA were reverse transcribed using a VN-anchored oligo-dT primer containing an 8-nt UMI (GTAATACGACTCACTATAG-NNNNNNNN-T30VN) and template-switching oligonucleotide (AAGCAGTGGTATCAACGCAGAGTACATrGrG+G). cDNA was pre-amplified for 14 cycles using in silico PCR primer (AAGCAGTGGTATCAACGCAGAGT) and 3′ primer (GTAATACGACTCACTATA), followed by AMPure XP bead purification. The cDNA was fragmented to approximately 300 bp using Tn5 transposase and processed using the TruePrep DNA Library Prep Kit V2 (Vazyme, TD501). Final libraries were amplified with Tn5 adaptor and 3′ primer (AATGATACGGCGACCACCGAGATCTACACTAGATCGCTCGTCGGCAGCGTCAGATGTGTATAAGAGACAGGTAATACGACTCACTATA) and sequenced as 150-bp paired-end reads on an Illumina Hiseq X Ten platform.

### SLIM-seq data analysis.

Raw sequencing data from all input and IP samples were processed by fastp, Kallisto, and tximport to quantify the gene-level abundance. To evaluate the relative m^6^A level of each gene, the fold-change between IP and input sample read counts was calculated by DEseq2, representing m^6^A enrichment level. High-confidence m^6^A-tagged genes were identified using the following criteria: TPM greater than 1 in input samples, log_2_(fold change) for IP/input greater than 0 with an adjusted *P* value of less than 0.05.

### Statistics.

The flow cytometry standard data were analyzed using FlowJo software. The ImageJ software was used for the analysis of band signal intensities. The statistical analysis was conducted using GraphPad Prism software. Comparisons between 2 groups were performed using an unpaired 2-tailed Student’s *t* test. Two-way ANOVA with Tukey’s post hoc test was used to calculate statistical significance among multiple groups. All data are presented as the mean ± SD. *P* values of less than 0.05 were considered statistically significant.

### Study approval.

All animal experimental protocols were reviewed and approved by the Institutional Animal Care and Use Committee of Zhengzhou University (approval ZZUIRB 2024-75).

### Data availability.

RNA-seq and m^6^A-seq datasets are available at Gene Expression Omnibus database under accession code GSE298935. Values for all data points in graphs are reported in the Supporting Data Values file.

## Author contributions

LZ and Huizhi Zhao designed experiments, performed research, analyzed the data, and drafted the Methods section of the manuscript. SW, Xueting Wu, DL, QY, YC, Xiuyun Wu, JZ, SZ, Hengchao Zhang, and Huan Zhang and Haojian Zhang performed research. QK analyzed the data and edited the manuscript. XQ, XA, and LC designed experiments, analyzed data, and wrote the manuscript.

## Conflict of interest

The authors have declared that no conflict of interest exists.

## Funding support

- Natural Science Foundation of China (grants 82000121 and 82370127, to XQ, 82370126, to H Zhao, 82370113, to QK, and 82400143, to SW).
- China Postdoctoral Science Foundation (grants 2020T130610 and 2019M662519, to XQ).
- Natural Science Foundation of Henan Province (grant 252300421125, to XQ).

## Supplementary Material

Supplemental data

Unedited blot and gel images

Supplemental table 1

Supplemental table 2

Supplemental table 3

Supplemental table 4

Supporting data values

## Version 1 03/10/2026

In-Press Preview

## Version 2 05/01/2026

Electronic publication
