# Supplementary material for: Methyltransferase complex subunit METTL3 maintains genome stability of erythroid cells via MTHFD1-mediated nucleotide biosynthesis
Source: J Clin Invest. 2026 Mar 10;136(9):e196578. doi: 10.1172/JCI196578 (PMC13132386; doi:10.1172/JCI196578)
Supplement: Supplemental data [file jci-136-196578-s274.pdf]

SUPPLEMENTAL METHODS

Genomic DNA extraction and PCR genotyping

Mouse tail was lysed in lysis buffer. Proteinase K (4 µL/mL) was added to the lysis buffer, and the mixture was incubated overnight at 55 °C. DNA was precipitated with isopropanol and washed with 75% ethanol. PCR amplification was performed using 2×Flash PCR MasterMix (Cwbio, CW3009M). The primers were used for the *EpoR-tdTomato-Cre* reaction WT-F: GTGTCCGATTCTGGCATCTCAACAGA; WT-R: TGCACCCACACTCACGCCATACATA; Mut-R: CCTCCTCGCCCTTGCTCAC. The PCR condition was: (1) 95°C, 5min. (2) 95°C, 20s; 65°C, 20s; 72°C, 20s; 35 cycles. (3) 4°C, hold. The primers were used for the *CD169-tdTomato-Cre* reaction WT-F: GACCTTTTGTGATCCAGGTTTGCTT; WT-R: TGAGCTTTGAAG AGAAAGGCAAAGG; Mut-R: CATGAACTCTTTGATGACCTCCTCG. The PCR condition was: (1) 95°C, 3min. (2) 95°C, 15s; 65°C, 20s; 72°C, 25s; 32cycles. (3) 72°C, 7min. (4) 4°C, hold. The *Mettl3*$^{fl/fl}$ primers were: primer F: AAAAGGCAATGTGCTTCTATGCCCG; primer R: TGCACGATGATAAAAGCCACTGT AAC. The PCR conditions were: (1) 94°C, 5min. (2) 94°C, 30s; 56.5°C, 30s; 68°C, 25s; 15 cycles. (3) 94°C, 30s; 56°C, 30s; 68°C, 25s; 25 cycles. (4) 68°C, 10 min.

Preparation of single-cell suspensions for flow cytometry

For mouse bone marrow cells, the femur and tibia were removed and placed in a dish containing Buffer I (PBS, 2% FBS, 2 mM EDTA), with the bone cavity rinsed into a 15 mL centrifuge tube using a 1 mL syringe, followed by gentle dispersion of cells using a pipette to avoid clumping, filtration through a 70 µm filter into a new 50 mL tube, and adjustment to a final volume of 15 mL.

Staining and flow cytometric analysis of hematopoietic stem and progenitor cells

For quantification of total bone marrow (BM) hematopoietic stem and progenitor cells (HSPCs) compartments, cells were first blocked with CD16/32 Fc-blocking antibody (eBioscience, #553142). They were then stained with the following biotinylated lineage markers: Biotin-Gr-1 (eBioscience, #13-5931-75),

Biotin-CD11b (eBioscience, #13-0112-75), Biotin-CD3e (eBioscience, #13-0031-75), Biotin-B220 (eBioscience, #103204), and Biotin-Ter119 (eBioscience, #13-5921-75), followed by incubation at 4°C for 30 minutes. After incubation, cells were washed with 1mL of Buffer I by centrifugation at 300 × g for 5 minutes. For LSK^+ ($Lin^-Sca\text{-}1^+Kit^+$) cell staining, cells were labeled with: APC-Cy7-conjugated anti-mouse CD117 (BioLegend, #105826), APC-conjugated anti-mouse CD34 (BioLegend, #119310), BV605-conjugated anti-mouse Sca-1 (BioLegend, #108134), BV421-conjugated anti-mouse CD135 (BioLegend, #135314), BV510-conjugated anti-mouse CD150 (BioLegend, #115929), PE-Cy7-conjugated anti-mouse CD48 (BioLegend, #103424), PerCP-conjugated streptavidin (BioLegend, #405213); For LSK^- ($Lin^-Sca\text{-}1^-Kit^+$) cell staining, the following antibodies were used: APC-Cy7-conjugated anti-mouse CD117 (BioLegend, #105826), APC-conjugated anti-mouse CD34 (BioLegend, #119310), BV605-conjugated anti-mouse Sca-1 (BioLegend, #108134), BV510-conjugated anti-mouse CD16/32 (BioLegend, #101333), PerCP-conjugated streptavidin (BioLegend, #405213), PerCP-conjugated anti-mouse CD41 (BioLegend, #133918), BV421-conjugated anti-mouse CD105 (BD, #562760), PE-Cy7-conjugated anti-mouse CD150 (BioLegend, #115914). All staining steps were performed at room temperature for 30 minutes in the dark. After incubation, cells were centrifuged at 300 × g for 5 minutes, resuspended in 300 μL of Buffer I, and stained with the viability marker 7-AAD (BD, #559925) (0.5 μg/mL) for 10 minutes in the dark. Finally, cells were transferred to flow cytometry tubes and analyzed using a BD LSRFortessa™ flow cytometer.

Western blotting

Erythroid cells were collected in 1.5 mL EP tubes and centrifuged at 300 × g for 5 minutes at 4°C. The cells pellet was resuspended in 1 × PBS and centrifuged again under the same conditions, then placed on ice. Cell lysis: a lysis buffer was prepared by adding 100 × protease inhibitor (Solarbio, P6731) and 50 × phosphatase inhibitor (Solarbio, P8993) to RIPA buffer (CWBIO, CW2333S). The mixture was thoroughly mixed and

kept on ice for 3~5 minutes. The RIPA mixture was then added to the cells, followed by vortexing every 10 minutes. Complete lysis was achieved by incubating the cells on ice for 30 minutes. After lysis, the samples were centrifuged at 14,000 rpm for 10 minutes at 4°C. The supernatant was transferred to a new EP tube, and the protein concentration was determined using the BCA method (APPLYGEN, P1511). Protein quantification (BCA Assay): for each sample, a working solution was prepared by mixing BCA reagent and Cu^{2+} at a 49:1 ratio (200 μL total volume per sample). A standard curve was generated using gradient-diluted standards, and samples were diluted 10-fold with ddH_2O. Then, 200 μL of the BCA working solution was added to each well, and the plate was incubated at 37°C for 30 minutes. Absorbance was measured at 562 nm, and the protein concentration was calculated. Protein denaturation and electrophoresis: 5×loading buffer was added to the protein lysate to obtain a 1×working solution. The mixture was boiled for 5 minutes. A PAGE protein gel (Epizyme, PG212, LK304, LK303) was prepared for electrophoresis. Proteins were transferred to a PVDF membrane, which was then blocked with 5% BSA for 1 hour at room temperature. Primary antibodies were diluted as follows: GAPDH mouse monoclonal antibody (Epizyme, LF205S, 1:5000), β-Actin recombinant mouse monoclonal antibody (Epizyme, LF201S, 1:5000), γ-H2AX (abcam, ab81299, 1:3000), pATM (Thermo Fisher, MA1-2020, 1:1000), ATM (CST, #2873, 1:1000), pATR (CST, #2853,1:1000) , ATR (CST, #13934, 1:1000), pCHK1 (CST, #2348, 1:1000), CHK1 (CST, #2360, 1:1000), MTHFD1 (Proteintech,10794-1-AP, 1:1000). α-globin (Proteintech, 14537-1-AP, 1:20000), β-globin (Abcam, ab214049, 1:20000), METTL3 (CST, #86132, 1:1000). The membrane and diluted antibodies were incubated overnight at 4°C. The next day, secondary antibodies were applied: goat anti-rabbit IgG-HRP (Epizyme, LF102,1:5000), goat anti-mouse IgG-HRP (Epizyme, LF101,1:5000). An ultrasensitive luminescence solution (APPLYGEN, W0001) was prepared by mixing 1 mL of liquid A and 1 mL of liquid B. The PVDF membrane was incubated with the solution in a dark room. Images were captured using an automated gel imager, and exposure was documented.

SUPPLEMENTAL FIGURES AND FIGURE LEGENDS

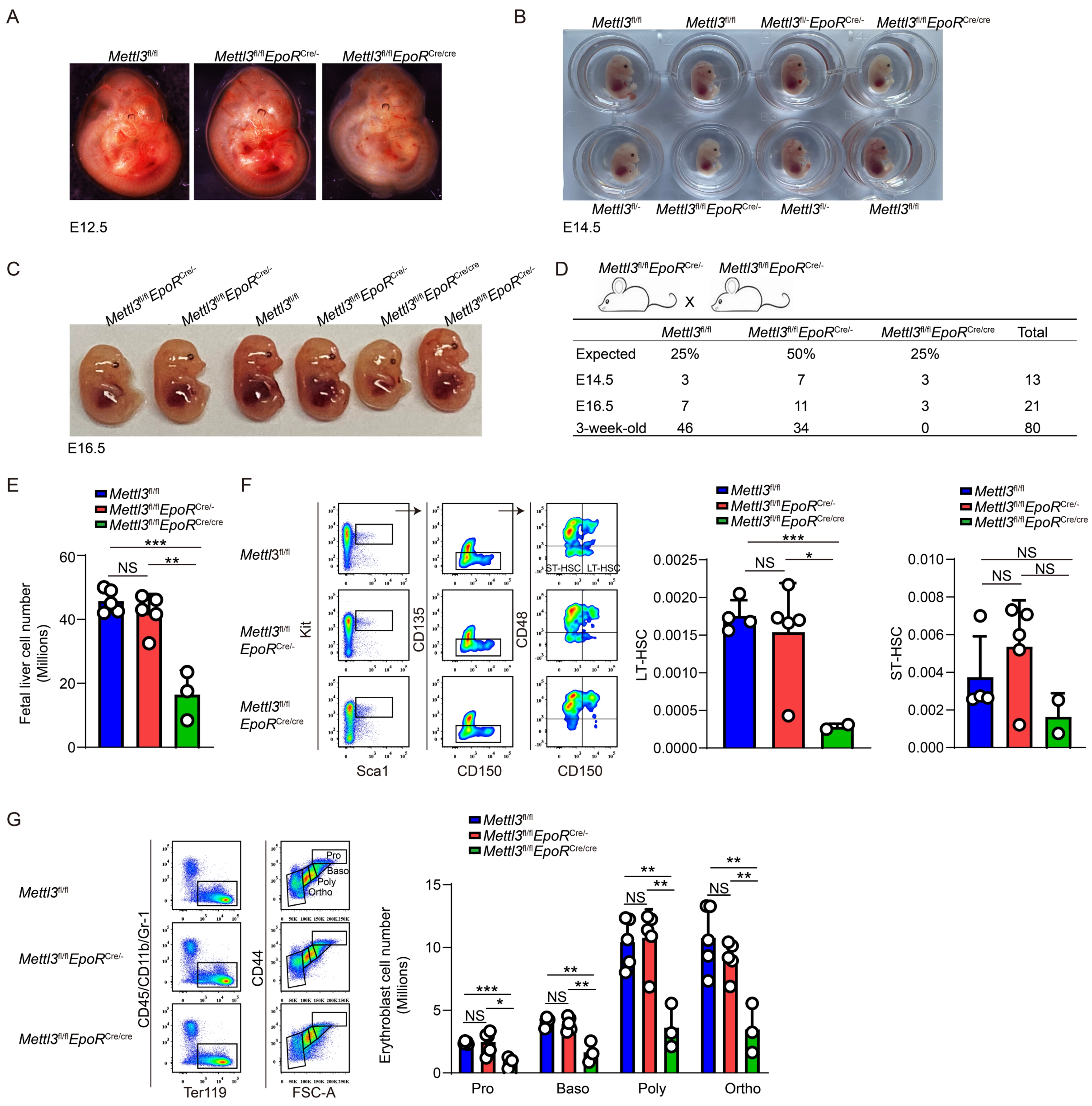

Supplementary Figure 1. Phenotypic analysis of *Mettl3*-deficient mouse embryos. (A) Representative photograph of *Mettl3*-deficient mouse embryos on E12.5 **(B)** E14.5 and **(C)** E16.5. **(D)** Mendelian ratio at the indicated stages. **(E)** Quantification of total fetal liver cells on E16.5 (n = 3-5/group). **(F)** Representative FACS plot and quantification analysis showing the percentage of LT-HSC, ST-HSC and **(G)** erythroid cells in E16.5 fetal liver (n = 2-5/group). Data were presented as mean ± SD. 2-way ANOVA with Tukey's post hoc test was used to calculate statistical significance among multiple groups. * $P < 0.05$, ** $P < 0.01$ and *** $P < 0.001$.

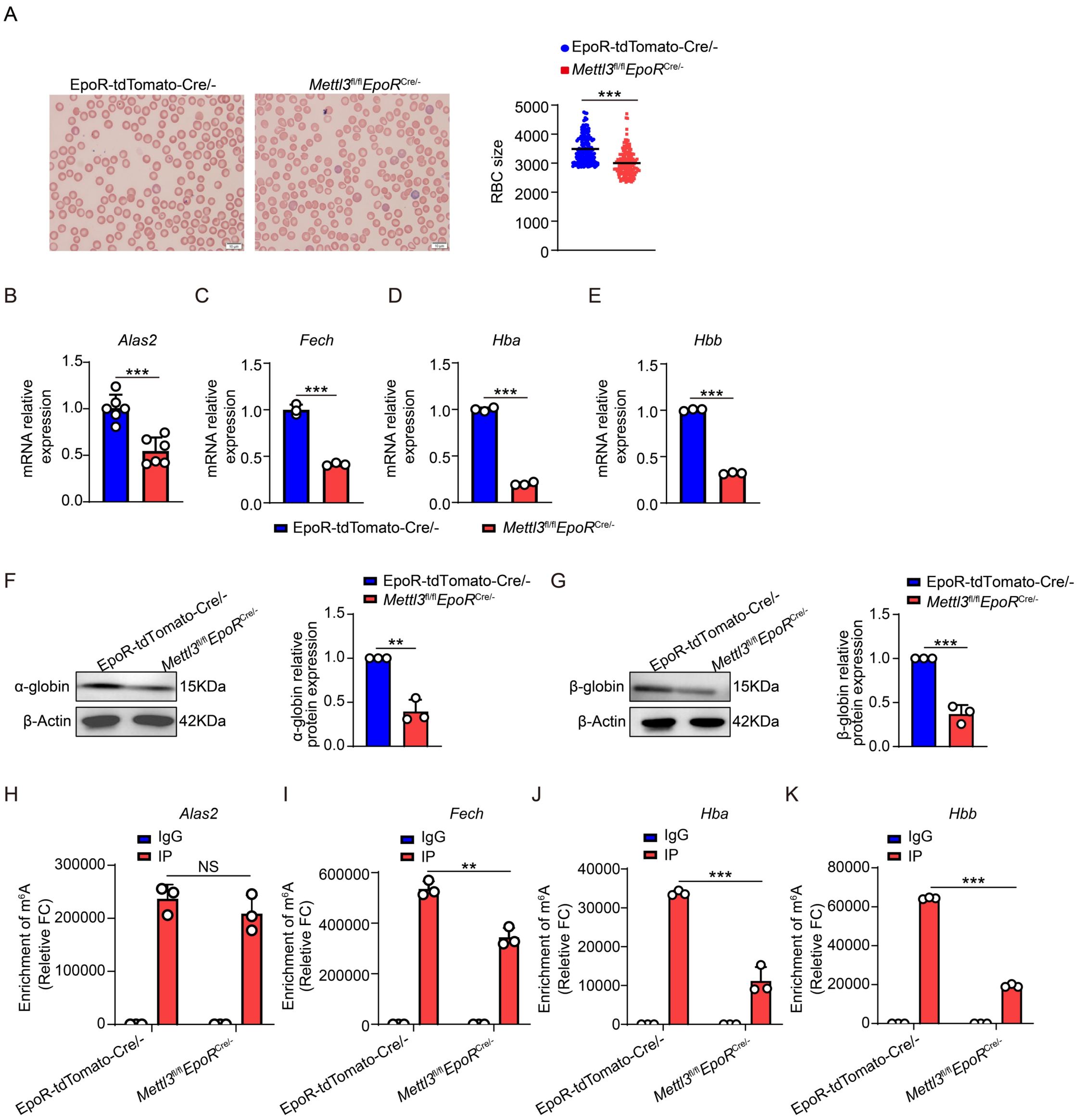

Supplementary Figure 2. *Mettl3* deficiency impairs hemoglobin synthesis in murine erythroblasts. (A) Staining of mouse erythrocyte blood smears and quantification of mice erythrocytes size. **(B-E)** qRT-PCR analysis of the expression of heme synthesis rate-limiting enzymes (*Alas2*, *Fech*) and globin genes (*Hba*, *Hbb*) in erythroblasts (n = 3-6/group). **(F)** Western blot analysis and quantification of α-globin protein levels in control and *Mettl3*-deficient murine erythroblasts (n = 3/group). **(G)** Western blot analysis and quantification of β-globin protein levels in control and *Mettl3*-deficient murine erythroblasts (n = 3/group). **(H-K)** Measurement of m^{6}A modification levels on *Alas2*, *Fech*, *Hba* and *Hbb* in murine erythroblasts by MeRIP-qPCR (n = 3/group). Data were presented as mean ± SD. Comparisons between two groups were performed using an unpaired two-tailed Student's t-test. 2-way ANOVA with Tukey's post hoc test was used to calculate statistical significance among multiple groups. ** $P < 0.01$ and *** $P < 0.001$.

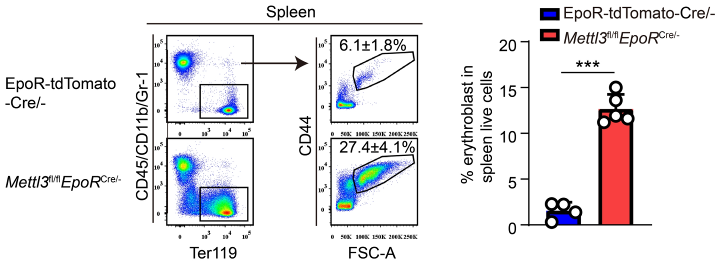

Supplementary Figure 3. Flow cytometric analysis of erythroid cells in control and *Mettl3* knockout mouse spleen. (n = 4-5/group). Data are presented as mean ± SD. Comparisons between two groups were performed using an unpaired two-tailed Student's t-test. *** $P < 0.001$.

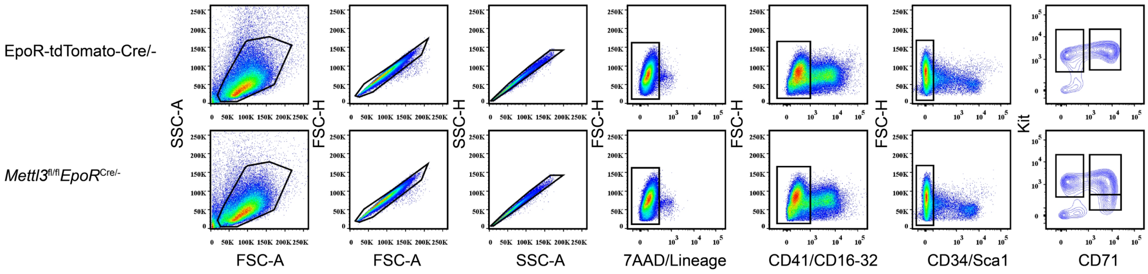

Supplementary Figure 4. Gating strategy for control and *Mettl3* knockout mice bone marrow erythroid progenitors.

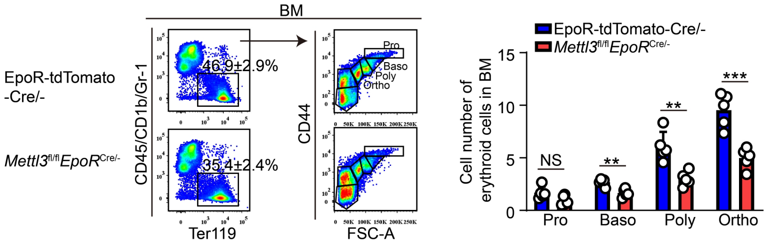

Supplementary Figure 5. Flow cytometric analysis of erythroid cells in control and *Mettl3* knockout mouse bone marrow. (n = 5/group). Data are presented as mean ± SD. Comparisons between two groups were performed using an unpaired two-tailed Student's t-test. ** $P < 0.01$ and *** $P < 0.001$.

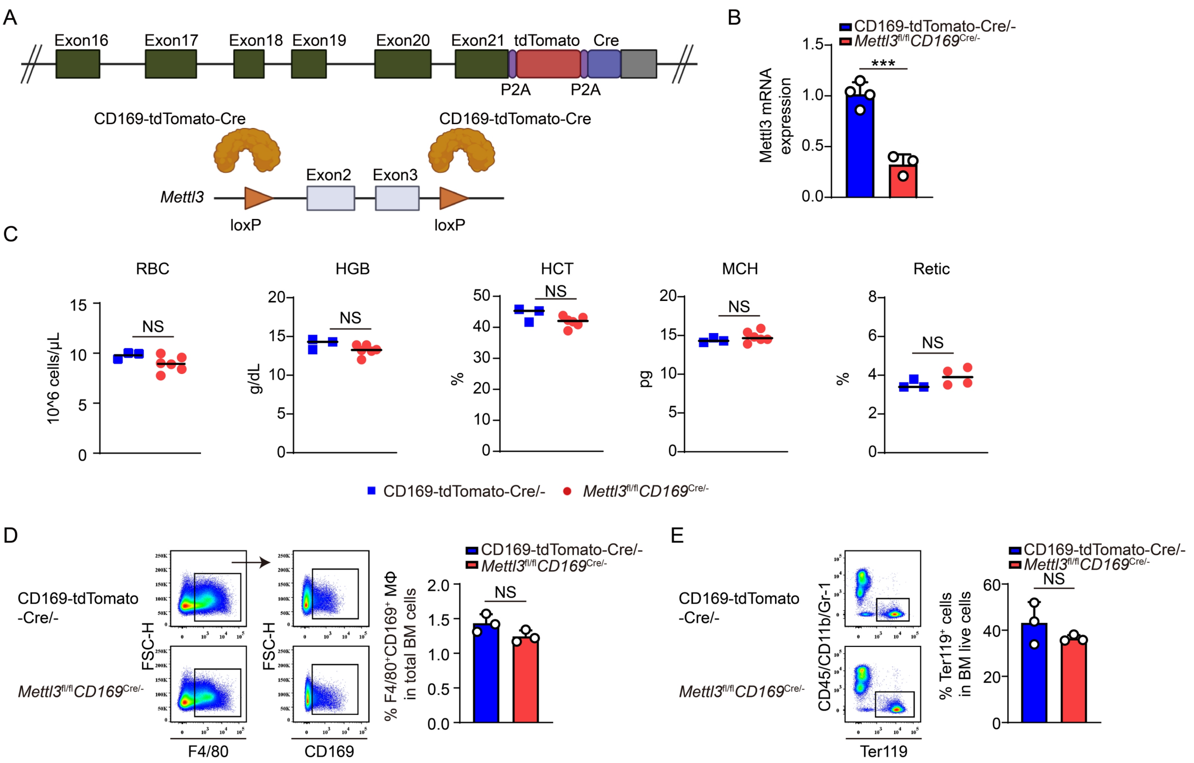

Supplementary Figure 6. Macrophage-specific *Mettl3* knockout shows no effects on erythropoiesis. (A) Genetic scheme for *Mettl3* floxed allele deletion via *CD169-tdTomato-Cre* recombinase. **(B)** Knockout efficiency of *Mettl3* in bone marrow erythroblastic island (EBI) macrophages (n = 3-4/group). **(C)** Peripheral blood parameters showing no differences between control and *Mettl3*-defient mice (n = 3-6/group). **(D)** Flow cytometric analysis of EBI macrophages percentage (n = 3/group). **(E)** Flow cytometric analysis of Ter119^{+} cells percentage (n = 3/group). Data are presented as mean ± SD. Comparisons between two groups were performed using an unpaired two-tailed Student's t-test. *** $P < 0.001$.

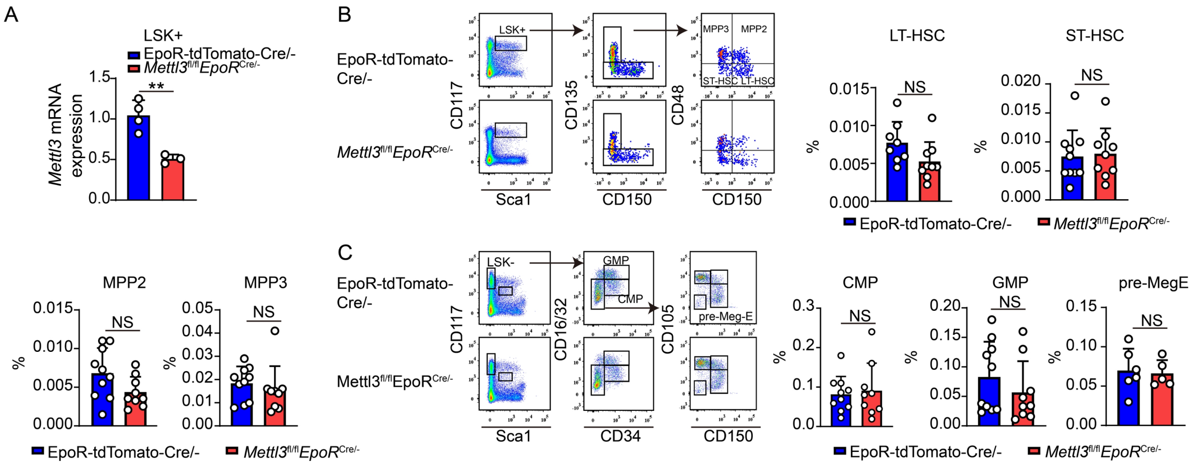

Supplementary Figure 7. *Mettl3* deficiency does not significantly alter the proportion of hematopoietic stem and progenitor cells in the bone marrow. (A) Expression levels of *Mettl3* in LSK^{+} cells from control and *Mettl3*-deficient mice by qRT-PCR and normalized to β-Actin (n = 3-4/group). **(B)** Flow cytometry gating strategy and the proportion of HSPCs compartments: Long-term hematopoietic stem cells (LT-HSCs), Short-term hematopoietic stem cells (ST-HSCs), Multipotent progenitor 2 (MPP2), Multipotent progenitor 3 (MPP3) gated on LSK^{+} population (Lin^{-}Kit^{+}Sca-1^{+}cells) (n = 8-10/group). **(C)** Representative flow cytometry plots for LSK^{-} lineage-committed progenitors: Common myeloid progenitors (CMPs), Granulocyte-macrophage progenitors (GMPs), Pre-megakaryocyte-erythroid progenitors (pre-MegEs) gated on LSK^{-} population (Lin^{-}Kit^{+}Sca-1^{-} cells) (n = 5-10/group). Data are presented as mean ± SD. Comparisons between two groups were performed using an unpaired two-tailed Student's t-test. ** $P < 0.01$.

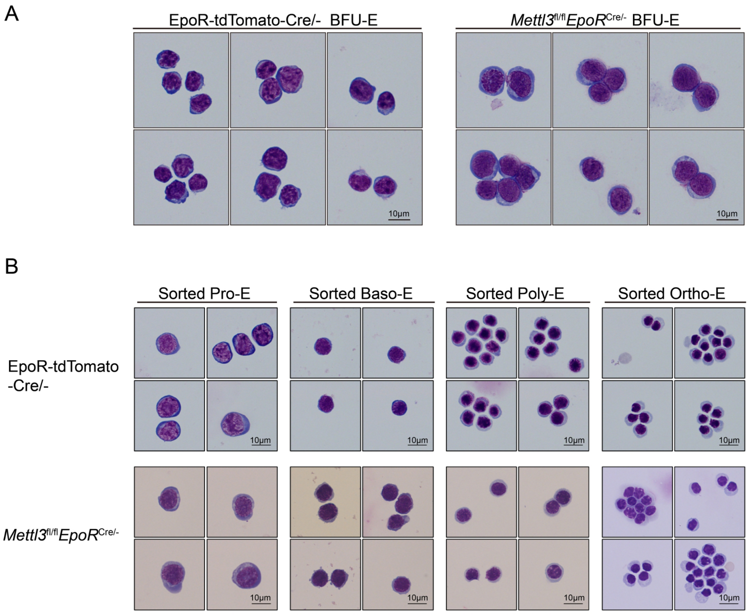

Supplementary Figure 8. Characterization of BFU-E and erythroblast cells in *Mettl3*-deficient mice. (A) Composite representative cytospin images of the sorted BFU-E cells from control and *Mettl3*-defient mice in the bone marrow. **(B)** Composite representative cytospin images of the sorted proerythroblasts (pro-E), basophilic erythroblasts (baso-E), polychromatic erythroblasts (poly-E), and orthochromatic erythroblasts (ortho-E) cells from control and *Mettl3*-defient mice. Scale bar: 10 μm.

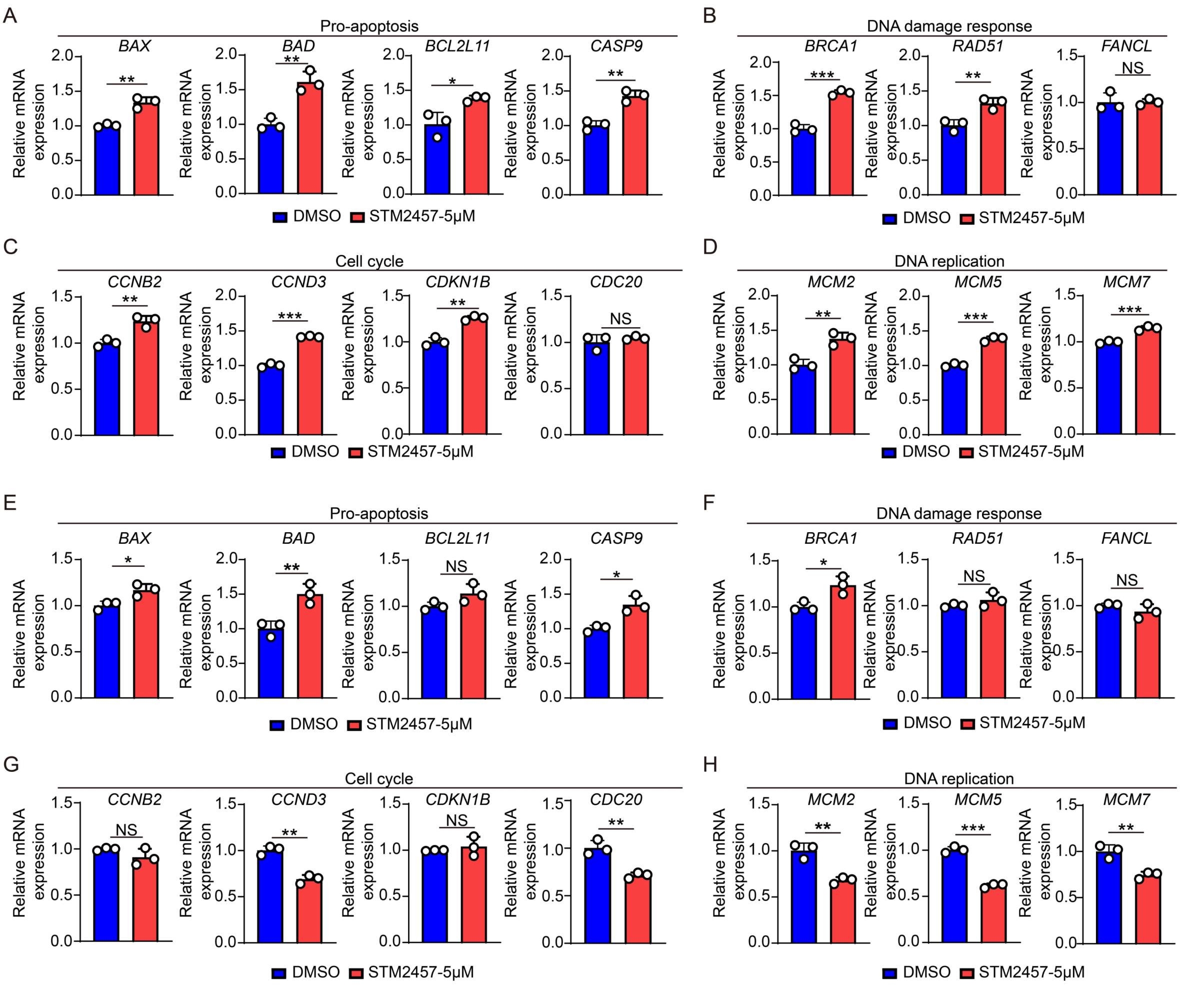

Supplementary Figure 9. Gene expression analysis of sorted human CFU-E and GPA⁺ cells (Pro-E) at day 7 of the in vitro culture. (A-D) The expression levels of pro-apoptosis, DNA damage response, cell cycle, and DNA replication genes were assessed by qRT-PCR and normalized to GAPDH in sorted human CFU-E cells at Day7 (n = 3/group). (**E-H)** Analysis of gene expression in sorted human GPA^{+} cells at Day7. Relative mRNA levels of pro-apoptosis, DNA damage response, cell cycle, and DNA replication genes were quantified by qRT-PCR and normalized to GAPDH (n = 3/group). Data are presented as mean ± SD. Comparisons between two groups were performed using an unpaired two-tailed Student's t-test. * $P < 0.05$, ** $P < 0.01$, *** $P < 0.001$.

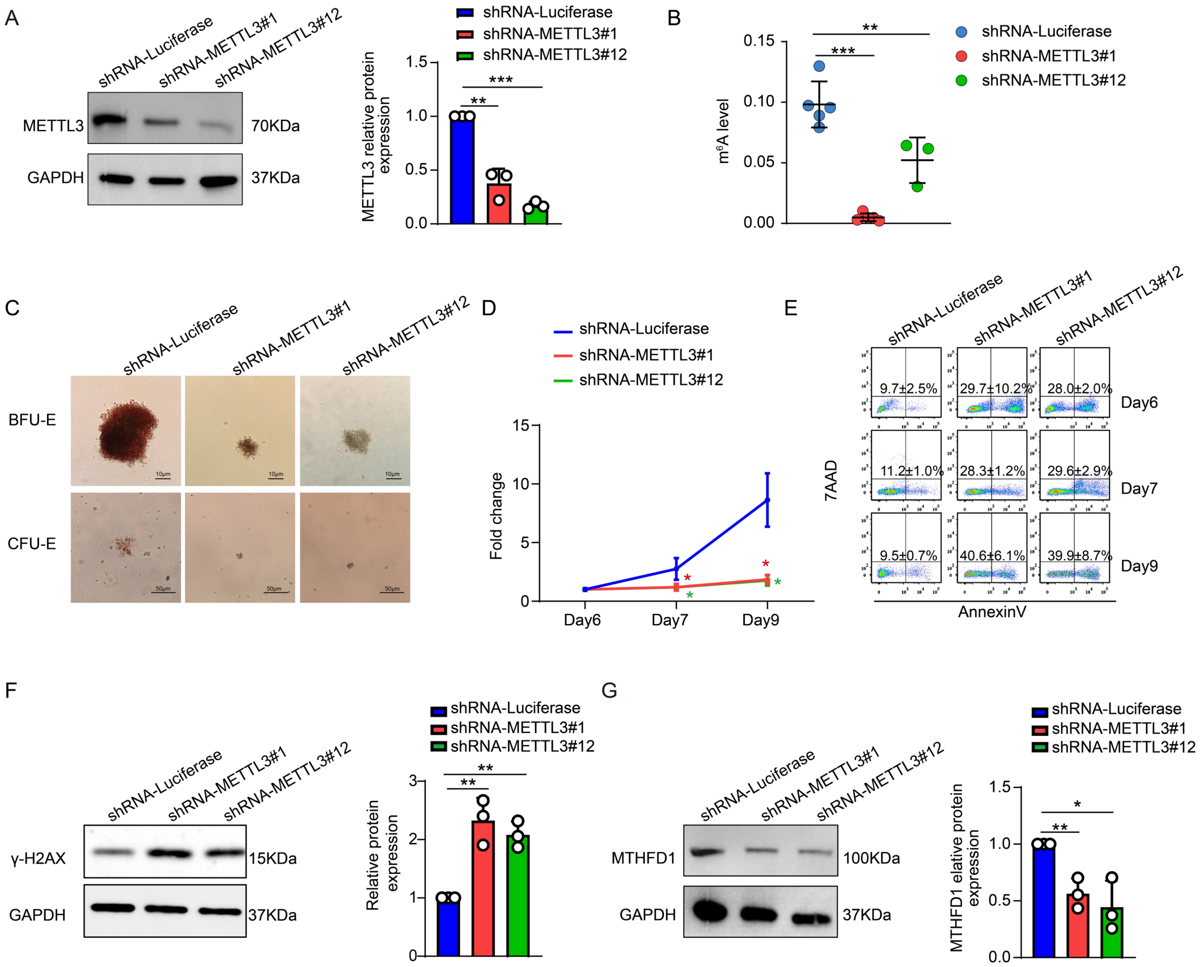

Supplementary Figure 10. Knockdown of *METTL3* in human erythroid cells impairs erythropoiesis. (A) Western blot analysis and quantification of METTL3 protein levels following knockdown (n = 3/group). **(B)** Global m^{6}A levels in total RNA of shRNA-Luciferase, shRNA-*METTL3*#1, shRNA-*METTL3*#12 transduced cells at day7 using colorimetric assay (n = 3-5/group). **(C)** Colony-forming ability of Luciferase control and *METTL3*-knockdown erythroid progenitor cells at Day6. **(D)** Growth curves of erythroid cells transduced with shRNAs targeting Luciferase or *METTL3* (#1 and #12) (n = 3/group). **(E)** Flow cytometric analysis of apoptosis in erythroid cells transduced with control or *METTL3*-targeting shRNAs at days 6, 7, and 9 using Annexin V/7AAD staining. **(F)** Western blot analysis and quantification of γ-H2AX protein levels in Luciferase control and *METTL3*-knockdown erythroid cells (n = 3/group). **(G)** Analysis and quantification of MTHFD1 protein expression in *METTL3*-knockdown erythroid cells (n = 3/group). Data were presented as mean ± SD. 2-way ANOVA with Tukey's post hoc test was used to calculate statistical significance among multiple groups. * $P < 0.05$, ** $P < 0.01$ and *** $P < 0.001$.

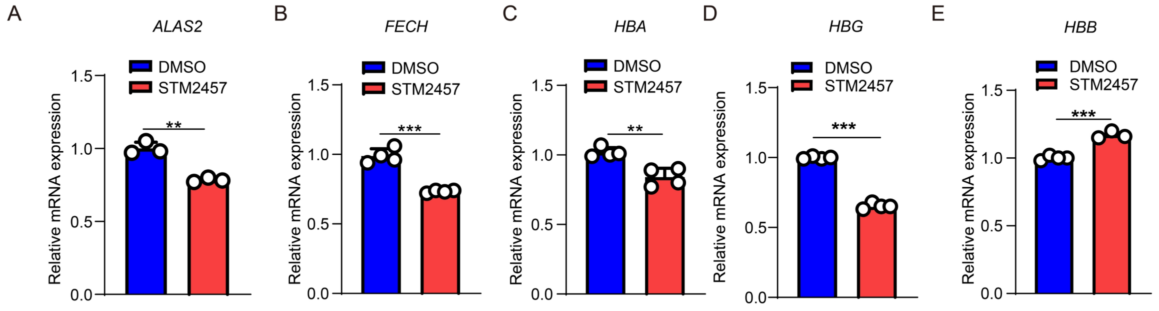

Supplementary Figure 11. Effects of STM2457 on human erythroid hemoglobin gene expression. (A-B) mRNA expression levels of heme synthesis genes (*ALAS2*, *FECH*) (n = 3-4/group). **(C-E)** mRNA expression levels of globin genes (*HBA*, *HBG*, *HBB*) (n = 3-4/group). Comparisons between two groups were performed using an unpaired two-tailed Student's t-test. ** $P < 0.01$, *** $P < 0.001$.

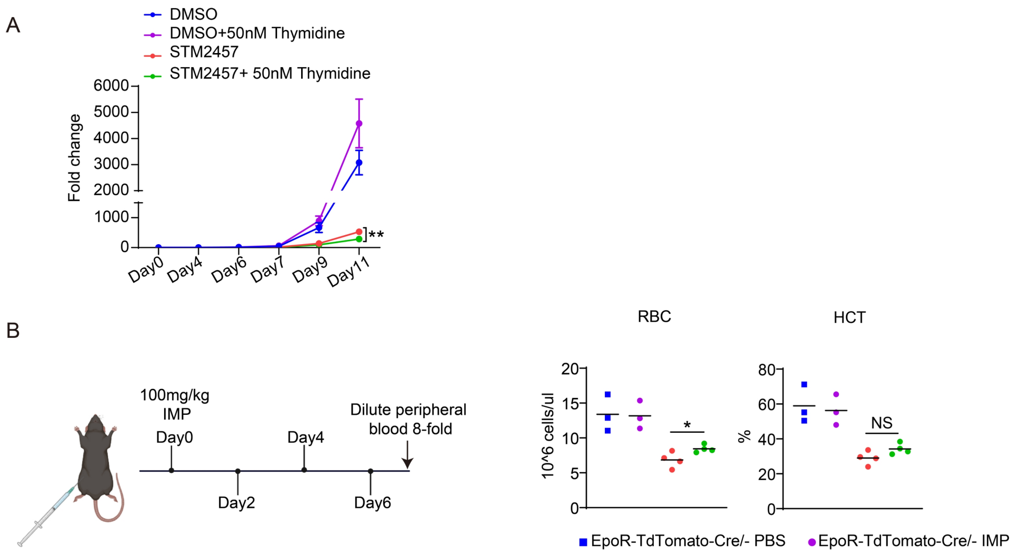

Supplementary Figure 12. Analysis of exogenous thymidine supplementation in vitro and IMP injection in mice. **(A)** Effect of in vitro thymidine supplementation on the proliferation of erythroid cells treated with DMSO or STM2457 (n = 3/group). **(B)** Recovery of red blood cells (RBC) and hematocrit (HCT) in mice following intraperitoneal injection of IMP. Data were presented as mean ± SD. 2-way ANOVA with Tukey's post hoc test was used to calculate statistical significance among multiple groups. * $P < 0.05$. ** $P < 0.01$.

Supplemental table S5. qRT-PCR primer and shRNA sequences

Name	Sequence 5'-3'	Note
Mettl3-F	CGTAGTGATAGTCCCGTGCC	qPCR primers for mice genes
Mettl3-R	TGGCGTAGAGATGGCAAGAC	
Mthfd1-F	GGGAATCCTGAACGGGAAACT	
Mthfd1-R	TGAGTGGCTTTGATCCCAATC	
Alas2-F	GCAGCTATGTTGCTACGGTC	
Alas2-R	GATGGGGCAGCGTCCAATAC	
Fech-F	CAGACAGATGAGGCTATCAAAGG	
Fech-R	CACAGCTTGTTGGACTGGATG	
Hba-F	TTGGCTAGCCACCACCCT	
Hba-R	CCAAGAGGTACAGGTGCA	
Hbb-F	TTAAGGCTCCTGGGCAATAT	
Hbb-R	TGCCAACAACTGACAGATGC	
β-Actin-F	CGTCGACAACGGCTCCGGCATG	
β-Actin-R	GGGCCTCGTCACCCACATAGGAG	
sh*METTL3*#1-F	GCCTTAACATTGCCCACTGAT	Human shRNA primer
sh*METTL3*#1-R	ATCAGTGGGCAATGTTAAGGC	
sh*METTL3*#12-F	CGTCAGTATCTTGGGCAAGTT	
sh*METTL3*#12-R	AACTTGCCCAAGATACTGACG	
sh*MTHFD1*#1-F	GCTGAAGAGATTGGGATCAAA	
sh*MTHFD1*#1-R	TTTGATCCCAATCTCTTCAGC	

Name	Sequence 5’-3’	Note
sh*MTHFD1*#5-F	CCAAGCGTTTCCTGGAGAAAT	Human shRNA primer
sh*MTHFD1*#5-R	ATTTCTCCAGGAAACGCTTGG	
MTHFD1-F	GTTGAAGGAGCAAGTACCTGG	qPCR primers for human genes
MTHFD1-R	GGTAGCTGCACTAAGAACCCA	
METTL3-F	TTGTCTCCAACCTTCCGTAGT	
METTL3-R	CCAGATCAGAGAGGTGGTGTAG	
BAX-F	CCCGAGAGGTCTTTTTCCGAG	
BAX-R	CCAGCCCATGATGGTTCTGAT	
BAD-F	CCCAGAGTTTGAGCCGAGTG	
BAD-R	CCCATCCCTTCGTCGTCCT	
BCL2L11-F	TAAGTTCTGAGTGTGACCGAGA	
BCL2L11-R	GCTCTGTCTGTAGGGAGGTAGG	
CASP9-F	CTTCGTTTCTGCGAACTAACAGG	
CASP9-R	GCACCACTGGGGTAAGGTTT	
BRCA1-F	GAAACCGTGCCAAAAGACTTC	
BRCA1-R	CCAAGGTTAGAGAGTTGGACAC	
RAD51-F	CAACCCATTTCACGGTTAGAGC	
RAD51-R	TTCTTTGGCGCATAGGCAACA	
FANCL-F	ATGAGGGATTCATCTCGGCTC	
FANCL-R	AGGTAGTGCATACAGCTCTTGT	
CCNB2-F	CCGACGGTGTCCAGTGATTT	

Name	Sequence 5'-3'	Note
CCNB2-R	TGTTGTTTTGGTGGGTTGAACT	qPCR primers for human genes
CCND3-F	TACCCGCCATCCATGATCG	
CCND3-R	AGGCAGTCCACTTCAGTGC	
CDKN1B-F	AACGTGCGAGTGTCTAACGG	
CDKN1B-R	CCCTCTAGGGGTTTGTGATTCT	
CDC20-F	GCACAGTTCGCGTTCGAGA	
CDC20-R	CTGGATTTGCCAGGAGTTCGG	
MCM2-F	CCGTGACCTTCCACCATTTGA	
MCM2-R	GGTAGTCCCTTTCCATGCCAT	
MCM5-F	ATGTCGGGATTCGACGATCCT	
MCM5-R	CCAGGTTGTAATGCCGCTTG	
MCM7-F	CCTACCAGCCGATCCAGTCT	
MCM7-R	CCTCCTGAGCGGTTGGTTT	
ALAS2-F	ACCTACCGTGTGTTCAAGACT	
ALAS2-R	AGATGCCTCAGAGAAATGTTGG	
FECH-F	GGAGATGTTCACGACTTCCTTC	
FECH-R	GAATGGTGCCAGCTTATTCTGA	
HBA-F	GACCTGCACGCGCACAAGCTT	
HBA-R	GCTCACAGAAGCCAGGAACTTG	
HBB-F	AGGAGAAGTCTGCCGTTACTG	

Name	Sequence 5'-3'	Note
HBB-R	CCGAGCACTTTCTTGCCATGA	qPCR primers for human genes
HBG-F	GGCAACCTGTCCTCTGCCTC	
HBG-R	GAAATGGATTGCCAAAACGG	
Mthfd1-3'UTR-WT sequence	TTAACTCTGTTTCCAGCCAAGTGGTGTTCAACAGAAGGACCTGCGCCACATCTGGAAGGAGTGAAGTTACTCTTGGTTCCGATAGTGTTTACACCATTTCTGCTGCTTATACTT	
Mthfd1-3'UTR-Mut sequence	TGCCCGCTGTTTCCAGCCAAGTGGTGTTCAACAGAAGGCCCTGCGCCACATCTGGAAGGAGTGAAGGGCCGCTTGGTTCCGATAGTGTGGCCCCCATTTCTGCTGCTTCGCCGT	
