# Supplementary material for: Methyltransferase complex subunit METTL3 maintains genome stability of erythroid cells via MTHFD1-mediated nucleotide biosynthesis
Source: J Clin Invest. 2026 Mar 10;136(9):e196578. doi: 10.1172/JCI196578 (PMC13132386; doi:10.1172/JCI196578)
Supplement: Unedited blot and gel images [file jci-136-196578-s275.pdf]

Full unedited blot for figures and supplemental figures

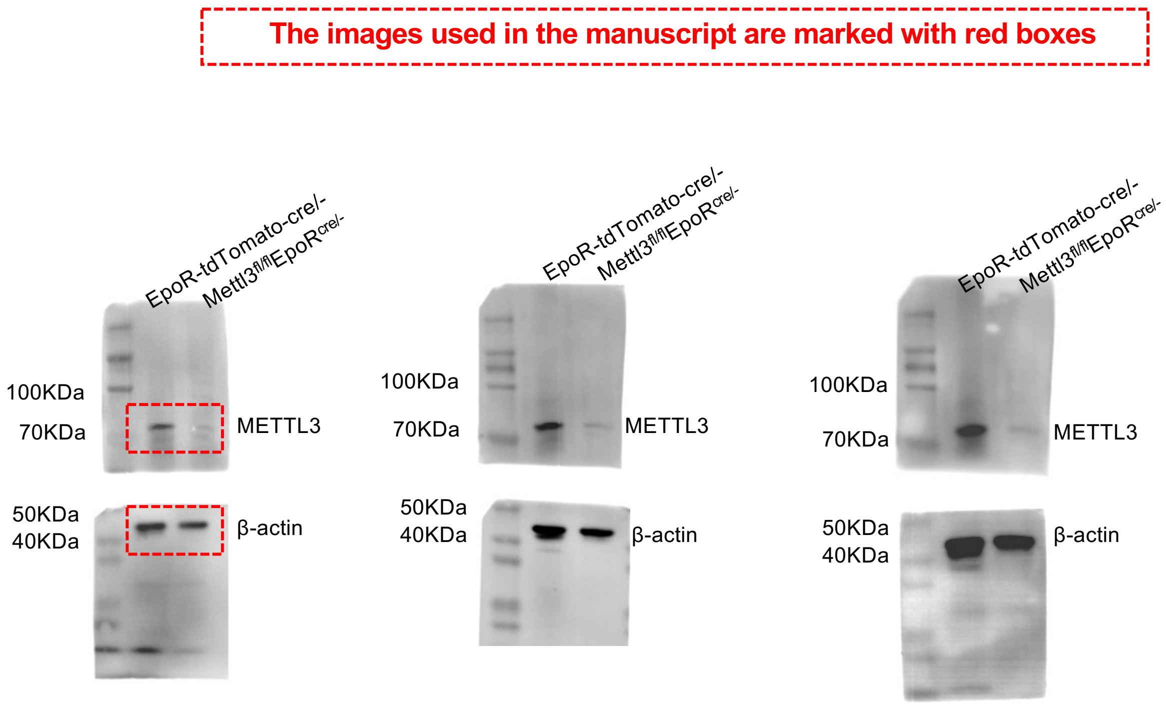

Full unedited blot for Figure 1C

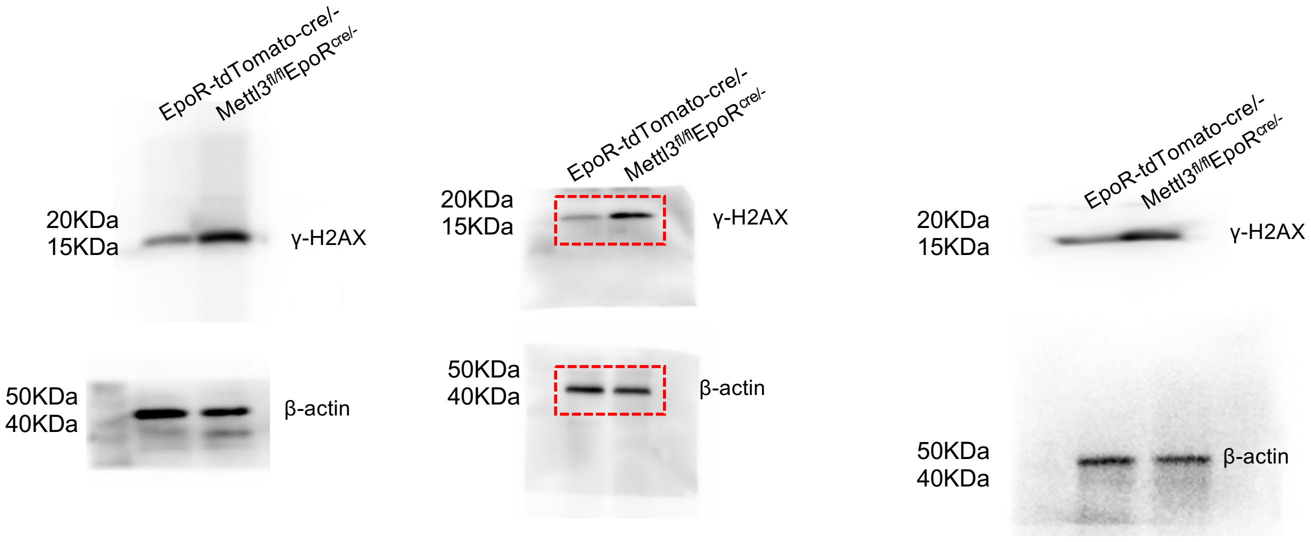

Full unedited blot for Figure 4M

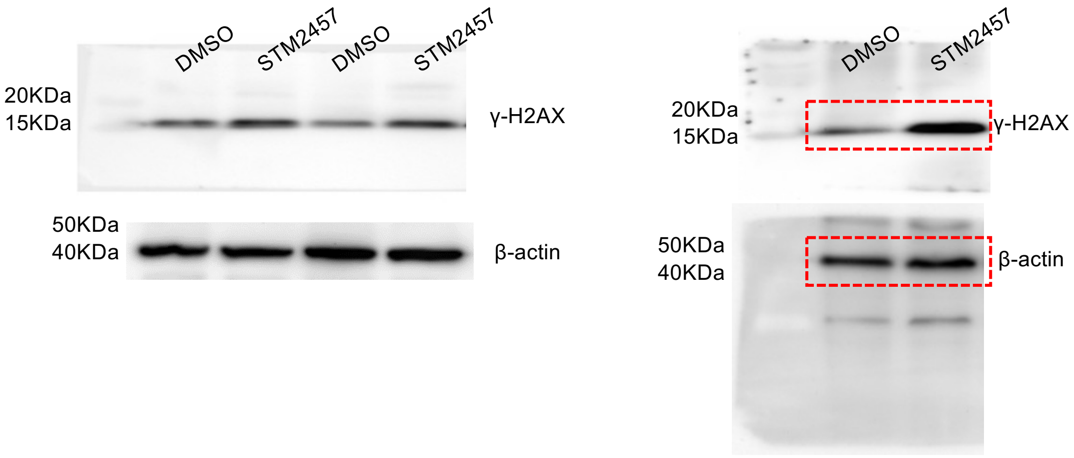

Full unedited blot for Figure 6J

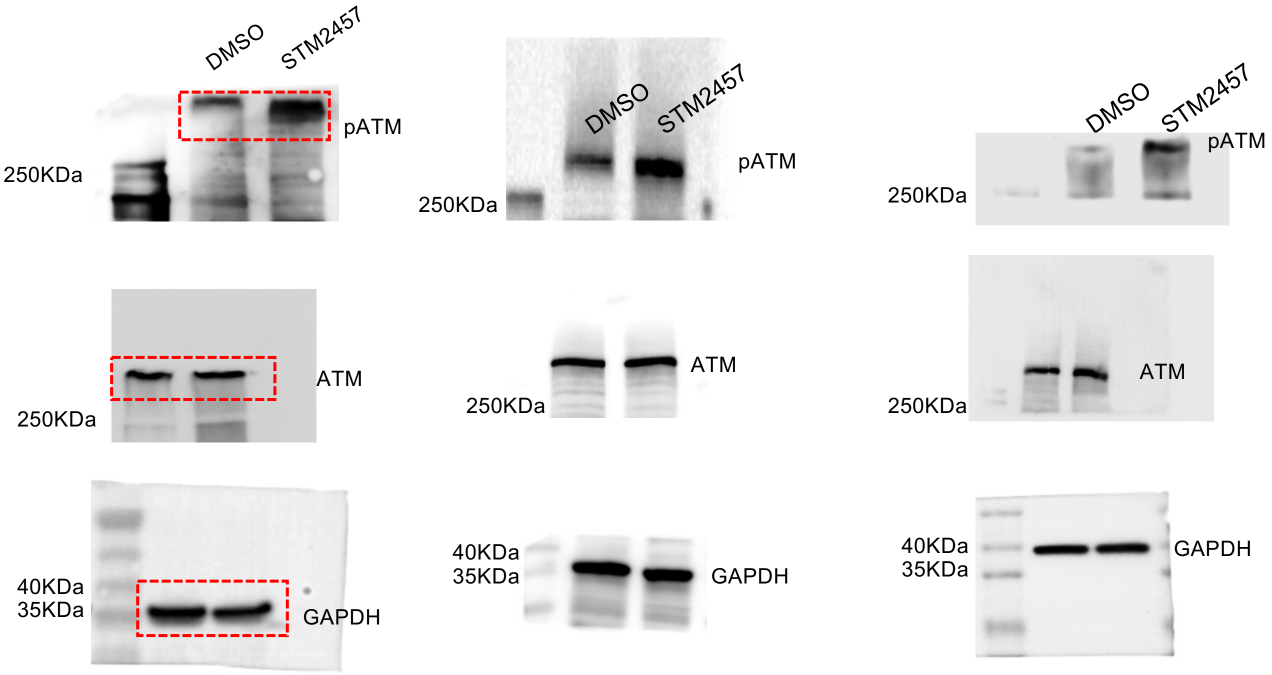

Full unedited blot for Figure 6J

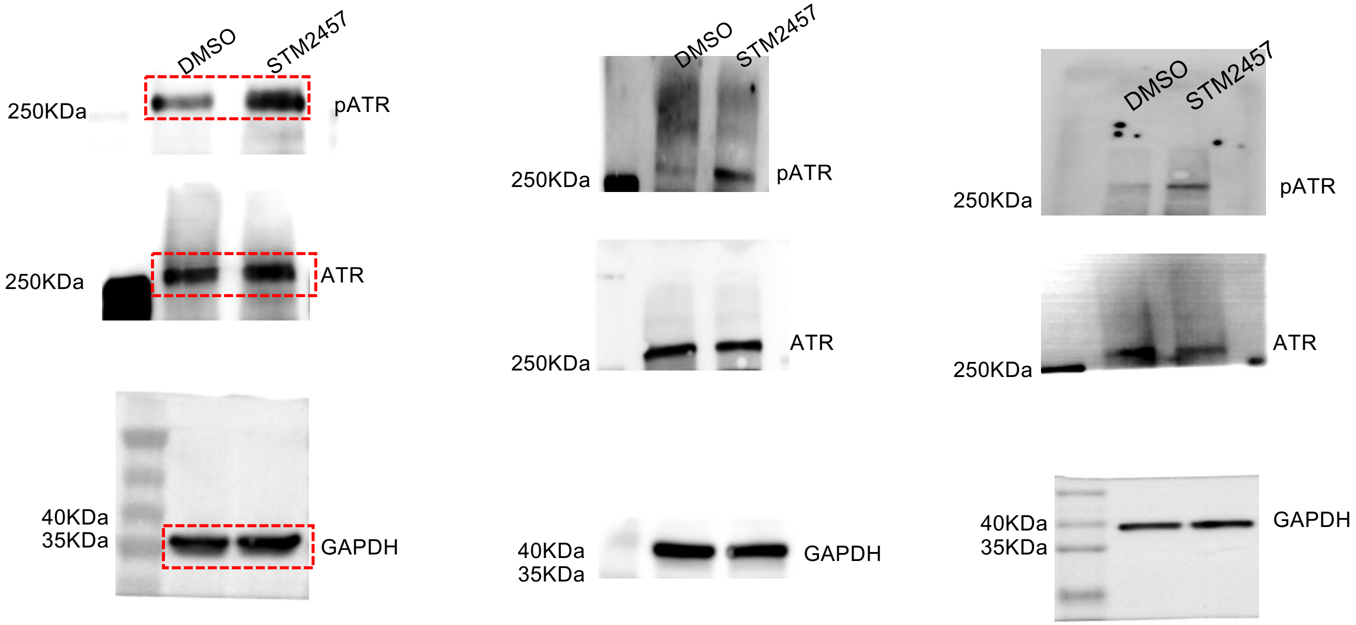

Full unedited blot for Figure 6J

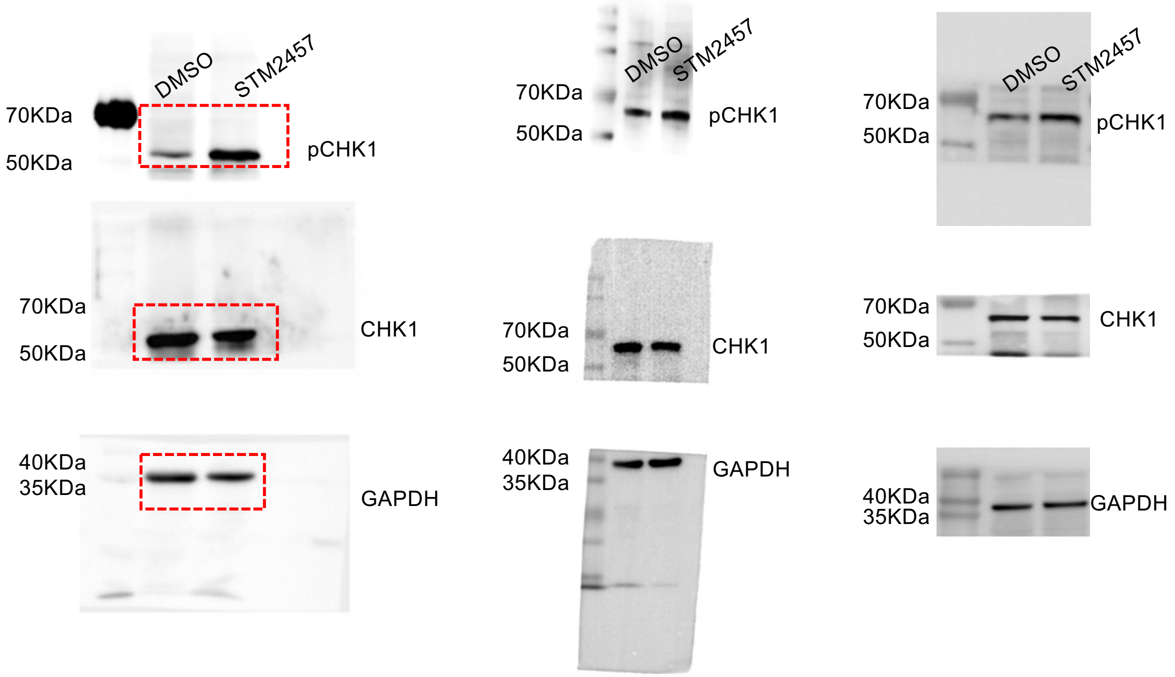

Full unedited blot for Figure 6J

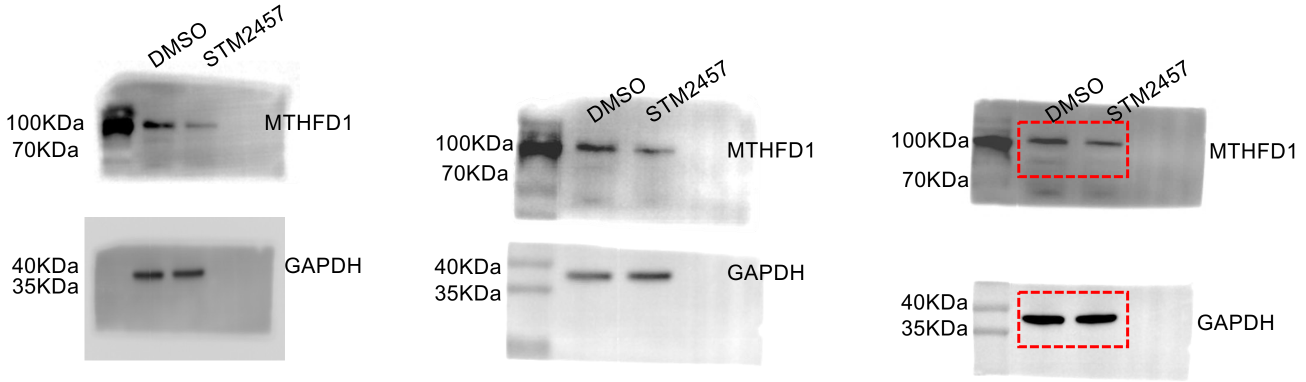

Full unedited blot for Figure 6L

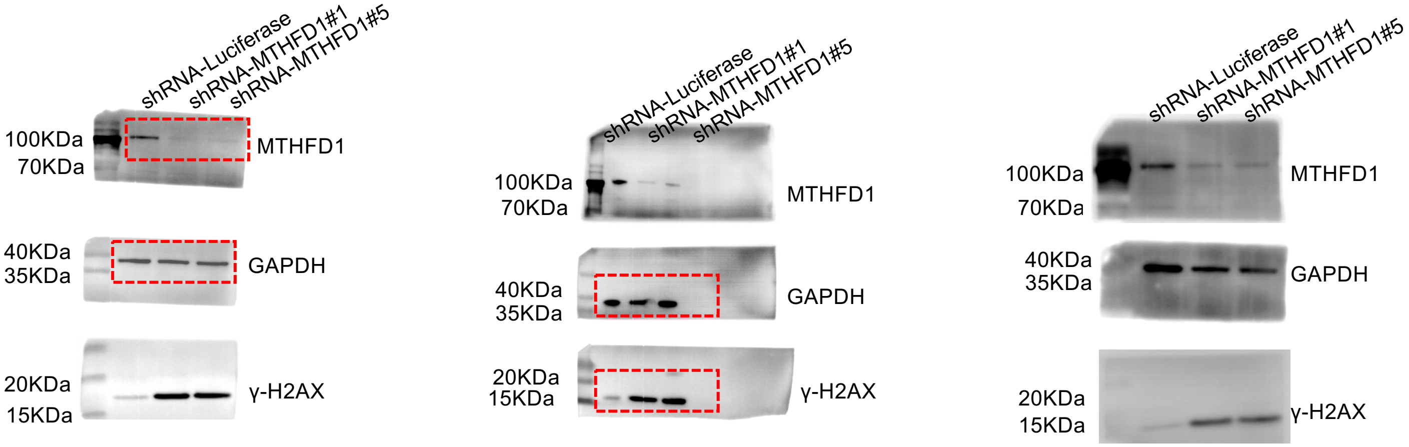

Full unedited blot for Figure 7C and Figure 7G

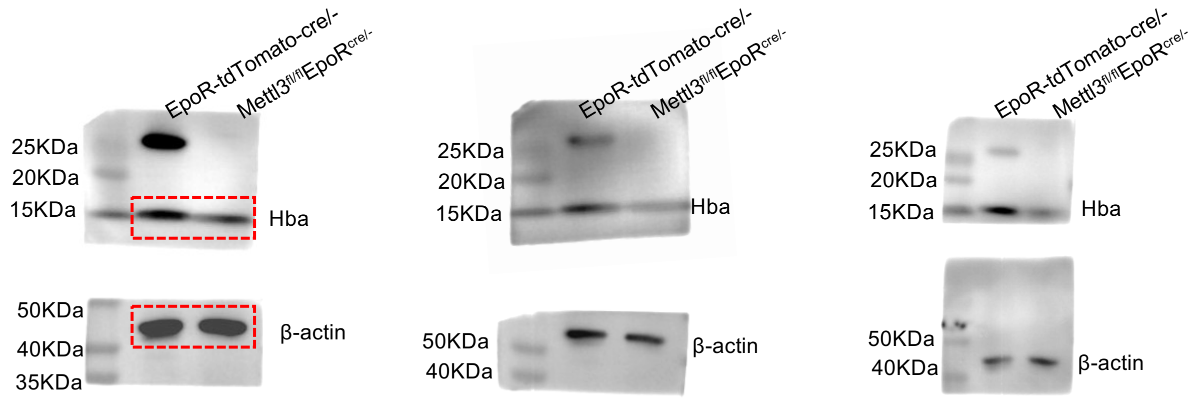

Full unedited blot for Supplementary Figure 2F

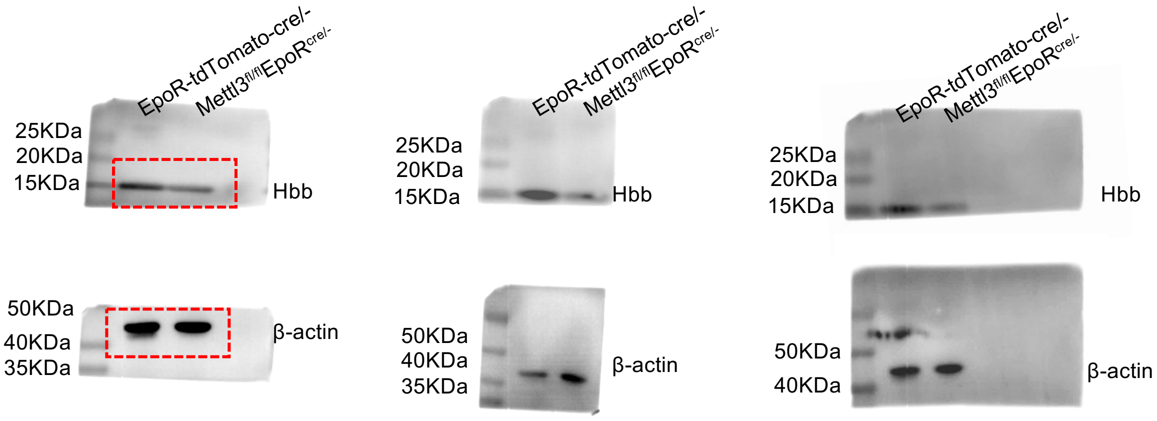

Full unedited blot for Supplementary Figure 2G

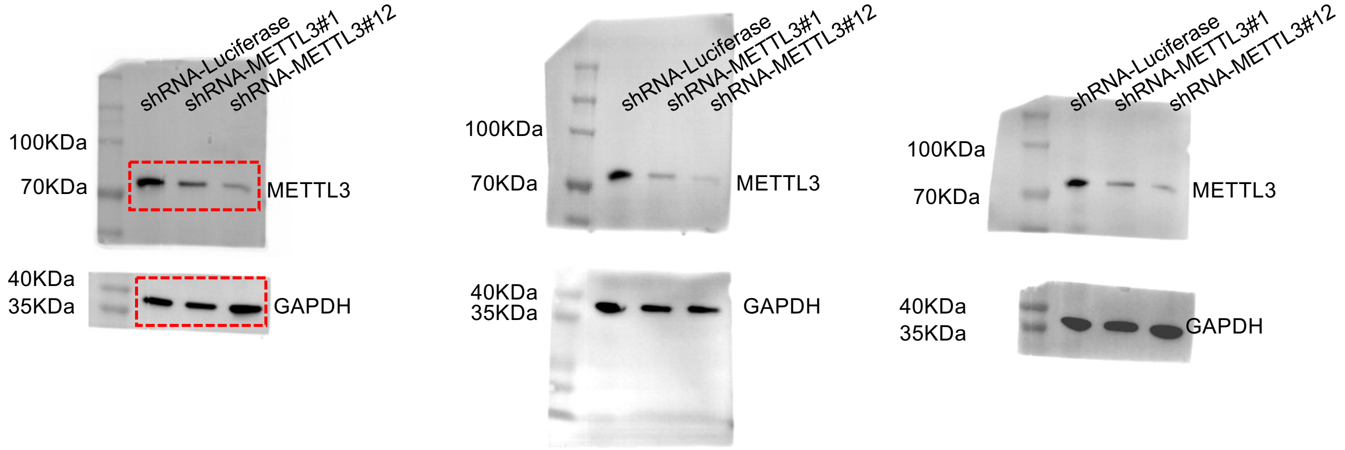

Full unedited blot for Supplementary Figure 10A

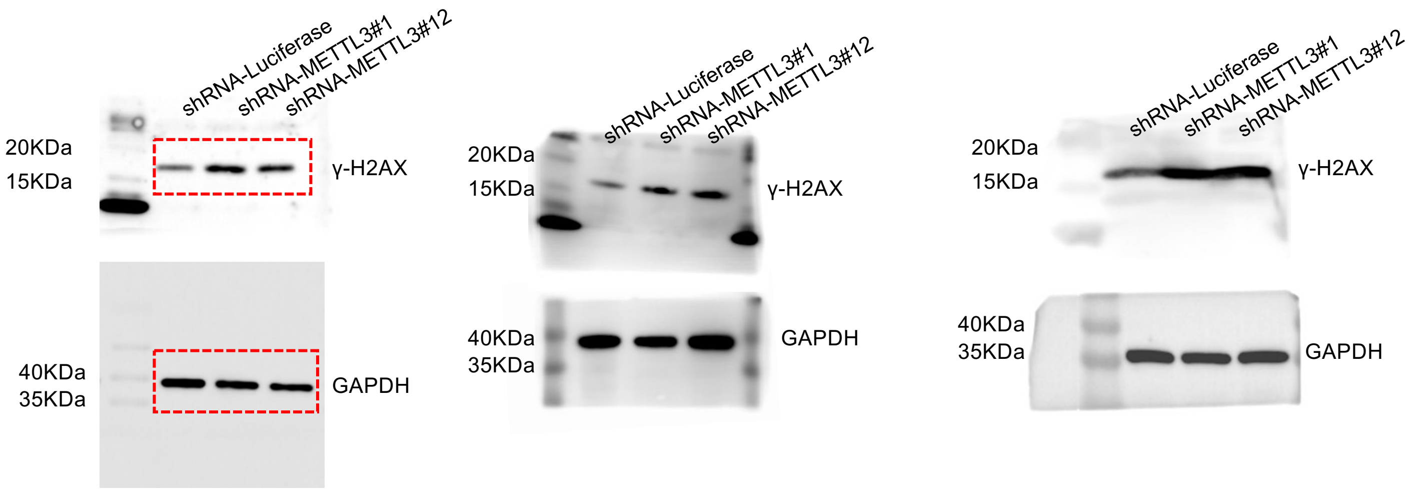

Full unedited blot for Supplementary Figure 10F

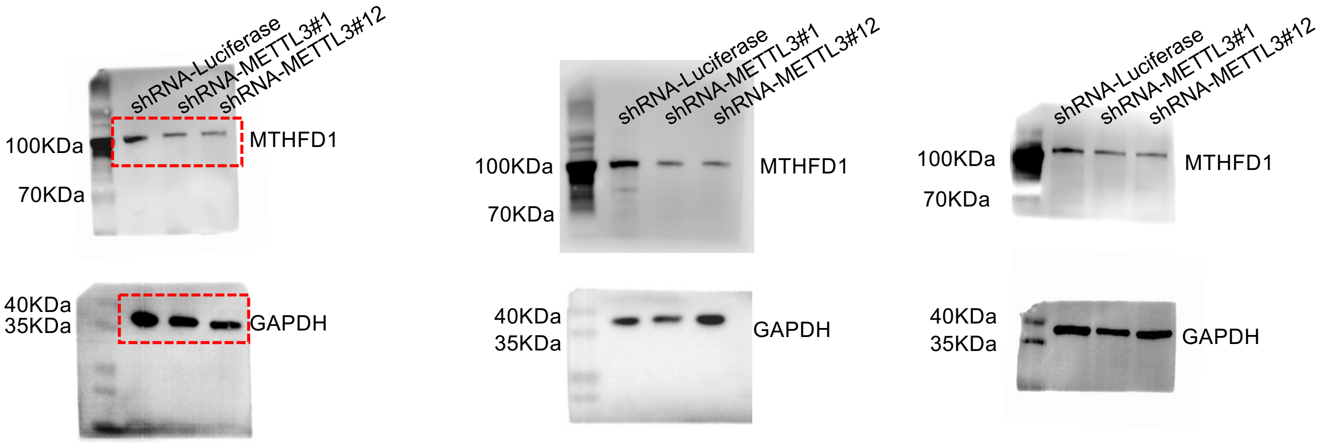

Full unedited blot for Supplementary Figure 10G
